# Integration of Microbial Metabolic Regulation and Abiotic Oxidation: Mechanisms of Artificial Humic Acid-Enhanced Hydroxyl Radical Generation in Paddy Soil

**DOI:** 10.34133/research.1269

**Published:** 2026-05-07

**Authors:** Shishun Wang, Taiping Zhang, Shuang Gai, Fan Yang, Kui Cheng, Zhuqing Liu

**Affiliations:** ^1^ School of Water Conservancy and Civil Engineering, Northeast Agricultural University, 150030 Harbin, China.; ^2^ International Cooperation Joint Laboratory of Health in Cold Region Black Soil Habitat of the Ministry of Education, 150030 Harbin, China.; ^3^ College of Engineering, Northeast Agricultural University, 150030 Harbin, China.

## Abstract

Hydroxyl radicals (•OH) generated under redox fluctuations in paddy soils are crucial for biogeochemical processes. Artificial humic acid (A-HA), as an emerging soil amendment, has been widely applied to improve soil quality. However, the impacts of A-HA on •OH generation and biogeochemical consequences remain under-explored. This study investigated the effects of A-HA on •OH generation, revealing that A-HA enhanced •OH accumulation from 43.93 to 142.30 μM upon oxygenation. The 0.5 M HCl-extracted Fe(II) was the dominant driver for •OH production via H_2_O_2_ as an intermediate. Multi-omics analyses demonstrated that A-HA reshaped the microbial community from a sulfate-reducing state to a copiotrophic state dominated by iron-reducing and fermentative bacteria. By acting as an electron shuttle and accelerating intermediate metabolism, A-HA overcame Fe(III) reduction kinetic barriers, providing a thermodynamic advantage that competitively suppressed sulfate-reducing bacteria. Additionally, •OH-induced mineralization and adsorption/fractionation by Fe(III)(oxyhydr)oxides decreased the content of dissolved organic matter (DOM). Fourier transform ion cyclotron resonance mass spectrometry confirmed that “saturated and oxidized” compounds were enriched during anaerobic incubation and subsequently preferentially degraded by •OH through dealkylation, decarboxylation, and oxygen addition, revealing a complementary pattern between anaerobic enrichment and oxidative degradation. Machine learning identified N/C, S/C, m/z, and O/C as key predictors of DOM transformation susceptibility, aligning with the metabolic enrichment of nitrogenous pathways. Furthermore, the enhanced •OH degraded propanil with a removal rate of up to 56.54%, suggesting that contaminants adsorbed on mineral surfaces were more susceptible to degradation. This study revealed a coupled mechanism in which microbial metabolic regulation during reduction enhances iron reduction for subsequent abiotic oxidation, providing new insights into microbial iron reduction, organic matter, and contaminant transformation in paddy ecosystems. Overall, these results further emphasized that A-HA substantially impacts paddy environments far beyond improving soil fertility.

## Introduction

Rice (*Oryza sativa*) serves as the staple food for over half of the global population, with a global cultivation area of 161 million hectares [1]. China dominates global rice production, contributing 30% of global total output and cultivating an extensive land area for the crop [2,3]. Paddy soil, as the prevalent anthropogenic wetland, is typically subject to flooding. Nevertheless, some paddy soils are susceptible to a cycle of flooding and drying due to the combined effects of irrigation, water evaporation, and terrain fluctuations [4]. Moreover, the implementation of noncontinuous flooding systems has led to notable advancements in rice-yield maintenance, irrigation water conservation, and greenhouse gas emissions reduction [5]. Consequently, this phenomenon has become increasingly prevalent throughout the rice cultivation period, resulting in an increased frequency of redox fluctuation in paddy soils [6,7]. The organic matter and iron species present in paddy soil can be reduced under conditions of flooding and subsequently react with O_2_ to produce reactive oxygen species (ROS) during the drying period [8,9]. Recently, several similar phenomena have been reported in diverse ecosystems, including coastal soils, river sediments, and wetland soils [8,10–12]. Given the annual increase in paddy cultivation areas and the development of noncontinuous irrigation management [13,14], paddy fields may be regarded as a source of soil ROS of comparable significance to the abovementioned ecosystems.

Reactive species induced by redox fluctuation primarily include hydroxyl radical (•OH), superoxide radical (O_2_^•−^), singlet oxygen (^1^O_2_), hydrogen peroxide (H_2_O_2_), and high-valent iron species [Fe(IV)]. Among these reactive species, •OH is generally considered the primary driver of the associated geochemical processes [15–20]. •OH (E_0_ ≈ 2.8 V) is the most reactive ROS and one of the strongest oxidizers in nature after fluorine, reacting with a wide range of chemicals nonselectively and at extremely high rates [21]. In soil environments, the generation of •OH is primarily driven by the oxygenation of low-crystallinity ferrous iron, with the accumulation of reactive Fe(II) serving as the rate-limiting step. Fe(II) accumulation is fundamentally governed by microbial dissimilatory iron reduction (DIR), the efficiency of which depends on microbial community composition and extracellular electron transfer performance. As the primary carbon source and electron shuttle, soil organic matter (SOM) has the potential to regulate microbial communities and electron transfer pathways. Meanwhile, given its ubiquity and abundance in soil, SOM constitutes the dominant reaction sink for •OH, and this nonselective oxidation alters the composition and properties of SOM, potentially affecting the subsequent DIR process. Therefore, elucidating the transformation of SOM is of great significance for understanding the generation of •OH under redox fluctuation conditions in paddy soil.

Artificial humic acid (A-HA) is a humic-like material that can be produced from waste biomass through hydrothermal humification, alkali-assisted hydrothermal treatment, catalytic pyrolysis, and related thermochemical humification processes, yielding products with SOM-like structures and abundant redox-active functional groups [22–26]. Recent studies have shown that A-HA and related humification products have broad application potential in contaminated soil remediation, saline–alkali soil improvement, carbon sequestration, fertilizer or phosphate adsorbent production, and functional carbon materials [17,27–31]. Together with its reported roles in supplementing SOM and regulating microbial communities, these findings indicate that A-HA not only is a soil amendment for improving soil quality but also has the potential to regulate microbial communities and alter soil redox processes. Furthermore, our previous studies have revealed that A-HA could reduce iron ions and alter iron oxide crystallinity, which further strengthens the link between A-HA and iron reduction [32,33]. Accordingly, the application of A-HA to paddy soil is predicted to exert a considerable impact on •OH generation under redox fluctuation, which in turn affects the transformation of SOM in paddy ecosystem.

Hence, this study investigated the effect of A-HA on •OH generation under simulating redox fluctuation in batch experiments. Specifically, (a) •OH accumulation and iron species formation under varying anaerobic times and A-HA concentrations were quantified. (b) The chemical mechanisms and pathways of •OH generation were determined through scavenging experiments. (c) The microbial mechanisms of the DIR process were elucidated through microbial multi-omics analyses. (d) The transformation characteristics of dissolved organic matter (DOM) during oxidation were elucidated, and the effects of A-HA and oxidation treatment on molecular characteristics of DOM were identified based on Fourier transform ion cyclotron resonance mass spectrometry (FT-ICR MS). Finally, the removal rates of typical organic contaminants were investigated during the oxidation process.

## Results and Discussion

### Accumulation of •OH

**Fig. 1.**
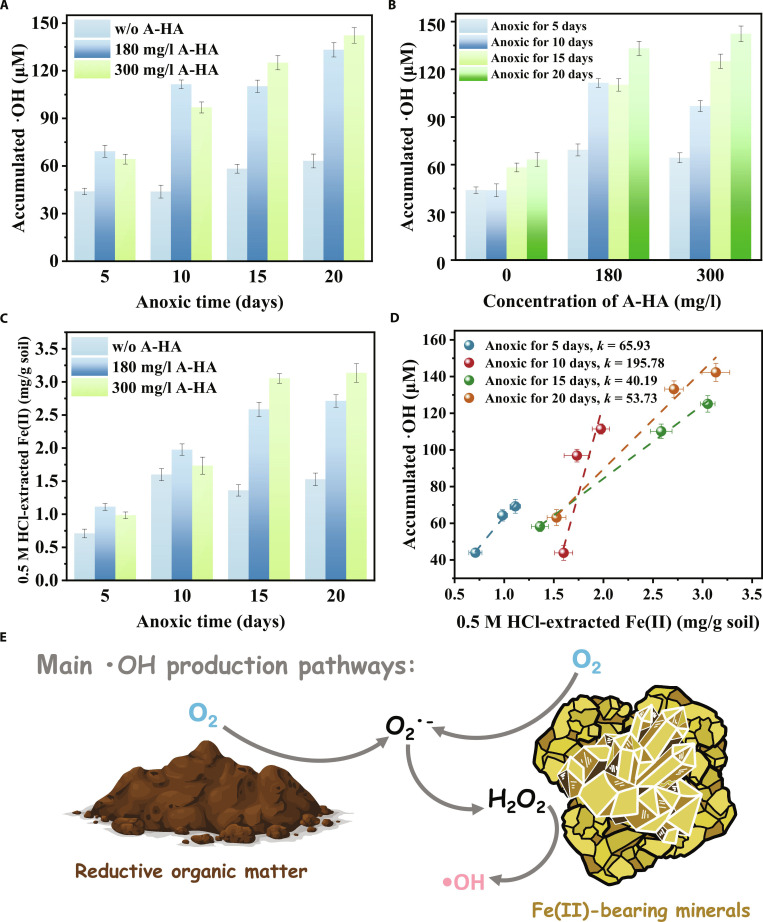
Concentration of accumulated •OH at different (A) anaerobic incubation times and (B) A-HA concentration. (C) Initial concentration of 0.5 M HCl-extracted Fe(II) after different anoxic incubation times. (D) Correlation between the accumulated •OH and the initial 0.5 M HCl-extracted Fe(II). (E) Mechanism diagram of main •OH production pathways.

Figure 1A demonstrates that soil suspensions after different anaerobic incubation days generated •OH at concentrations ranging from 43.93 to 142.30 μM within 12 h of oxygen exposure. By comparison, •OH generation was undetectable in suspensions that were not anaerobically incubated or were not exposed to oxygen (Fig. S1), which confirmed that the combination of these 2 conditions was critical factor in •OH production. Chen et al. [7] confirmed that dissolved oxygen is a prerequisite for the generation of •OH in anaerobically incubated soil. Furthermore, Fig. 1A and B illustrates that accumulated •OH increased from 43.93 to 63.14 μM, 69.25 to 133.16 μM, and 64.31 to 142.30 μM in soil suspensions with 0, 180, and 300 mg/l A-HA, respectively, as the anaerobic incubation period increased from 5 to 20 d. Normalized to soil mass, the corresponding •OH accumulations ranged from 109.83 to 157.85 μmol/kg soil, 173.13 to 332.90 μmol/kg soil, and 160.78 to 355.75 μmol/kg soil, respectively, which is consistent with previous studies on •OH accumulation in paddy soils [34,35]. Consequently, an increased accumulation of •OH was observed with both the addition of A-HA and the extension of anaerobic incubation. Specifically, suspensions with 180 mg/l A-HA exhibited maximum •OH accumulation during anaerobic incubation for 5 and 10 d, whereas suspensions with 300 mg/l A-HA demonstrated peak •OH accumulation during anaerobic incubation for 15 and 20 d (Fig. 1A). These results could be attributed to the combined effects of reduced iron and reduced organic matter, with detailed mechanisms to be elaborated in subsequent sections. Furthermore, •OH accumulation increased substantially within the first 2 h following oxygen exposure and then stabilized, with no notable variation observed after 4 h (Fig. S1). Both Li et al. [36] and Chen et al. [7] observed similar results in redox fluctuation incubations of magnetite and paddy soil, respectively. A possible explanation for this phenomenon is that the reaction between reducing substances and oxygen predominantly occurred on minerals or soil surfaces.

### Variations of Fe species

Reduced Fe species serve as the primary mediators driving the conversion of O_2_ to •OH. Specifically, upon oxygen exposure, reduced Fe species can reduce O_2_ either directly to H_2_O_2_ or stepwise via O_2_^•−^ as an intermediate, followed by dismutation to H_2_O_2_. The generated H_2_O_2_ then reacts with adjacent reduced Fe species to produce •OH [37]. Moreover, numerous studies have reported that surface Fe(II) species of low-crystallinity iron minerals and/or dissolved Fe(II), rather than high-crystallinity iron minerals, could be the predominant determinants of •OH accumulation [7,9,38]. Figure S3 and Fig. 1C illustrate the concentrations of dissolved Fe(II) and 0.5 M HCl-extracted Fe(II) during 8 h of oxygen exposure. The dissolved Fe(II) concentration remained below 0.02 mg/g soil and exhibited no observable correlation with oxygen exposure time, suggesting that the role of dissolved Fe(II) in the accumulation of •OH was negligible. In contrast, the 0.5 M HCl-extracted Fe(II) species concentration [representing surface Fe(II) concentration on low-crystallinity minerals] decreased during 8 h of oxygen exposure (Fig. S4). For anaerobic incubation times of 5, 10, 15, and 20 d, the Fe(II) fraction decreased from 1.11 to 0.40 mg/g soil, 1.97 to 0.59 mg/g soil, 3.05 to 0.54 mg/g soil, and 3.13 to 0.61 mg/g soil, respectively (Fig. S4). The decrease was rapid during the first 2 h of oxygen exposure and then leveled off in the subsequent 6 h. Furthermore, the initial 0.5 M HCl-extracted Fe(II) concentration in samples with 180 mg/l A-HA peaked at 5 and 10 d of anaerobic incubation, while samples with 300 mg/l A-HA peaked at 15 and 20 d (Fig. 1C). The control group without A-HA consistently exhibited the lowest concentration. Importantly, these trends were consistent with the accumulation of •OH, suggesting that 0.5 M HCl-extracted Fe(II) was the dominant factor driving •OH generation during oxygen exposure. In addition, the correlation between the initial 0.5 M HCl-extracted Fe(II) and the •OH accumulation was presented in Fig. 1D. The relationship was linear for all treatment groups, with slopes of 65.93, 195.78, 40.19, and 53.73 for anaerobic incubation times of 5, 10, 15, and 20 d, respectively. Notably, the slope at 10 d was higher than at other times, suggesting that additional reducing substances beyond Fe(II) contributed to •OH generation at this stage. This could be attributed to the accumulation of reducing organic matter during the early anaerobic phase, which has been demonstrated to activate O_2_ and generate •OH [39,40]. At 5 d, the limited anaerobic duration restricted the microbial reduction of organic matter, resulting in insufficient accumulation of reducing moieties. At 10 d, the reducing organic matter reached its maximum accumulation, serving as a supplementary electron donor for O_2_ activation alongside Fe(II) and thereby elevating the apparent slope. As anaerobic incubation progressed to 15 and 20 d, the continued microbial decomposition and consumption of these reducing organic compounds diminished their contribution to •OH generation. Therefore, 0.5 M HCl-extracted Fe(II) could be considered the dominant factor in •OH generation, while reducing organic matter served as a supplementary contributor during the early anaerobic stage.

### Chemical mechanisms of •OH generation

#### Effect of oxidation reduction potential

Figure S5A demonstrates the oxidation reduction potential (ORP) of anaerobically incubated soil suspensions prior to oxygen exposure, which represented the cumulative redox level of all matrices present in the soil suspension. Both increased anaerobic incubation days and A-HA addition resulted in a decrease in ORP. The measured ORP demonstrated linear relationships with the •OH accumulation and the initial 0.5 M HCl-extracted Fe(II) (Fig. S5B and C). However, the correlation coefficients associated with these linear relationships varied across different anaerobic incubation days, suggesting that factors beyond the ORP also governed these interactions.

#### Effect of Fe(II) species and H_2_O_2_

To quantify the contribution of Fe(II) species to the generation of •OH, 20 mM 2,2'-dipyridyl (BPY) was introduced into the oxidation process to scavenge the reaction of Fe(II) and O_2_. As demonstrated in Fig. S6A and B, the accumulated •OH decreased by 17.37%, 72.61%, and 74.22% with the addition of 0, 180, and 300 mg/l A-HA, respectively. These findings suggested that Fe(II) species were primarily responsible for the generation of •OH, while substances other than Fe(II) (e.g., reducing organic matter and microbial ROS) acted as secondary contributors. These results suggested that the enhanced •OH accumulation by A-HA was predominantly mediated through Fe(II)-driven pathways.

In addition, catalase (CAT) was applied to scavenge H_2_O_2_ for further validation of •OH generation mechanisms. As shown in Fig. S6A and C, the accumulated •OH decreased by 43.87%, 59.16%, and 74.75% with the addition of 0, 180, and 300 mg/l A-HA, respectively, indicating that H_2_O_2_ served as the primary intermediate for •OH formation. Interestingly, •OH remained detectable following H_2_O_2_ quenching, potentially attributed to other oxygen reduction pathways [41]. Furthermore, given that the molecular weight of CAT typically exceeds 200 kDa, H_2_O_2_ generated at the mineral interfaces cannot be fully decomposed by CAT due to steric hindrance. Consequently, this localized H_2_O_2_ persisted and continued to react with Fe(II) species to produce •OH. In summary, the chemical mechanism of •OH generation was predominantly driven by the reaction of low crystalline Fe(II) and O_2_, with H_2_O_2_ serving as a primary intermediate (Fig. 1E).

Additionally, it should be noted that in soil systems amended with exogenous organic substances, Fe(III) reduction can proceed through both direct abiotic pathways (chemical reduction by redox-active moieties) and indirect biotic pathways mediated by DIR. Although A-HA contains redox-active functional groups capable of directly reducing Fe(III) minerals, extensive evidence from analogous systems suggests that the abiotic contribution to total iron reduction is substantially lower than the biotic contribution. For example, Huang et al. [9] demonstrated that the abiotic reduction of Fe(III) by pyrogenic carbon accounted for only ~20% of total iron reduction in paddy soil. Similarly, Kappler et al. [42] and Xu et al. [43] reported that the direct chemical reduction of iron minerals by biochar was considerably less significant than microbially mediated pathways. Given the comparable redox-active moieties shared by A-HA and biochar, the abiotic contribution of A-HA to Fe(III) reduction is expected to be secondary to microbial processes in paddy ecosystem. Therefore, the following sections primarily focus on elucidating the microbial mechanisms underlying the enhanced DIR process in A-HA-amended systems.

### Microbial mechanisms of iron reduction

#### Microbial diversity and community structure

**Fig. 2.**
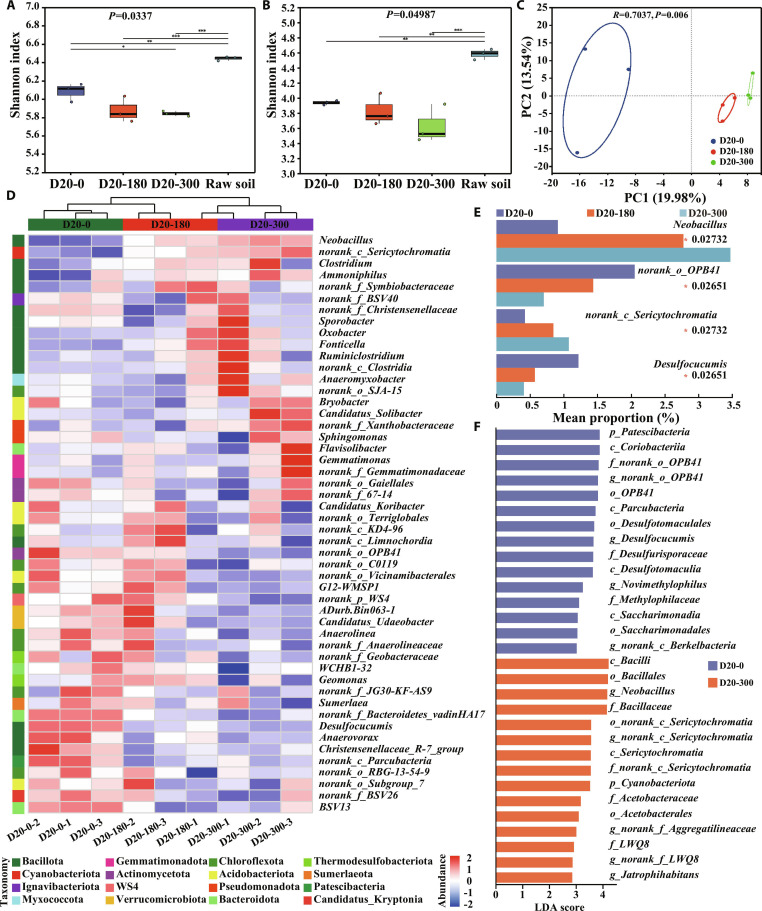
Shannon index of (A) bacterial and (B) fungal communities. (C) PCA and (D) genus-level heatmap of bacterial communities in D20-0, D20-180, and D20-300. (E) Genus-level differential abundance comparison of bacterial communities among D20-0, D20-180, and D20-300 by Kruskal–Wallis *H* test. (F) LDA score plot of LEfSe analysis of bacterial communities among D20-0, D20-180, and D20-300.

Bacterial and fungal communities after 20 d of anaerobic incubation were characterized to elucidate the role of microorganisms in A-HA-regulated DIR processes. The rarefaction curves of the Chao index reached asymptotes across all samples (Fig. S7), indicating sufficient sequencing depth for community analyses. The Chao index demonstrated that both bacterial and fungal richness decreased after anaerobic incubation, with the lowest richness generally observed in A-HA-amended systems and the highest richness in raw soil (Fig. S8). Similarly, the Shannon index revealed that the bacterial and fungal diversity decreased after anaerobic incubation and further declined with A-HA amendment (Fig. 2A and B). Principal components analysis (PCA) revealed a clear separation of microbial communities among different treatments (Fig. 2C and Fig. S9). Both bacterial and fungal communities exhibited pronounced differentiation in response to varying A-HA concentrations and anaerobic incubation, indicating that A-HA reshaped the microbial community during anaerobic incubation. The beta nearest taxon index (βNTI) indicated that bacterial community assembly was dominated by homogeneous selection (deterministic processes, βNTI < −2), likely driven by the strong environmental filtering of A-HA, whereas fungal assembly was primarily governed by stochastic processes (|βNTI| < 2; Fig. S10) [44].

Figure 2D and Fig. S11A and C illustrate that the bacterial community at the phylum level was dominated by *Bacillota*, *Bacteroidota*, *Actinomycetota*, *Acidobacteriota*, *Chloroflexota*, *Pseudomonadota*, and *Myxococcota* in A-HA-amended groups. The fungal community was dominated by *Ascomycota*, *Basidiomycota*, *Fungi_phy_Incertae_sedis*, and *Chytridiomycota* across all samples (Fig. S11B and D). At the genus level, the Venn diagrams in Fig. S12A and B identified 213 shared bacterial and 117 shared fungal genera. Specifically, the bacterial community in anaerobically incubated groups was characterized by *Flavisolibacter*, *Anaeromyxobacter*, *Candidatus_Koribacter*, *norank_f_Symbiobacteraceae*, and *WCHB1-32* (Fig. S12C). For fungi, *Leuconeurospora*, *Trichocladium*, *Talaromyces*, *Pseudeurotium*, and *Pseudogymnoascus* became the dominant genera under anaerobic conditions (Fig. S12D).

#### Taxonomic composition and functional shifts

The Kruskal–Wallis *H* test was employed to further identify the bacterial phyla and genera with relatively high abundance and significant differences among groups. At the phylum level, with increasing A-HA concentration, the abundance of *Thermodesulfobacteriota* decreased, while *Cyanobacteriota* increased (Fig. S13). At the genus level, with the increase of A-HA concentration, the abundance of *Neobacillus* and *norank_c_Sericytochromatia* increased, whereas the abundance of *norank_o_OPB41* and *Desulfocucumis* decreased (Fig. 2E). This indicated that A-HA amendment significantly suppressed sulfate-reducing bacteria and oligotrophic taxa while selectively enriching functional taxa capable of complex organic matter degradation, thereby driving the community function toward A-HA hydrolysis and subsequent iron reduction. Specifically, the decline in *Thermodesulfobacteriota* and *Desulfocucumis* with A-HA amendment signified a fundamental shift in terminal electron-accepting processes. In the absence of A-HA, although Fe(III) reduction is thermodynamically more favorable than sulfate reduction, the reduction of Fe(III) minerals was kinetically constrained by limited redox mediators for extracellular electron transfer [45,46]. This kinetic limitation rendered Fe(III) minerals less accessible as electron acceptors, thereby favoring the utilization of readily available sulfate by sulfate-reducing bacteria. The addition of A-HA altered this competitive landscape by directly serving as an electron shuttle and/or by regulating microorganisms to produce electron shuttles [47,48]. These electron shuttles overcame the kinetic barrier of Fe(III) mineral reduction, enabling the thermodynamic advantage of DIR to be realized. Consequently, iron-reducing taxa gained a competitive advantage in energy conservation, allowing them to proliferate more rapidly and outcompete sulfate-reducing genera for shared electron donors and carbon substrates [49,50]. In addition, the Kruskal–Wallis *H* test for fungal communities revealed that *Exophiala* emerged exclusively in A-HA-amended groups and increased with concentration, while *Rhodotorula* was enriched in D20-180 compared to D20-0 but was completely suppressed in D20-300 (Fig. S14). This indicated that the fungal community generally exhibited lower responsiveness to A-HA compared to bacteria, with only specific taxa demonstrating sensitivity to A-HA.

#### Identification of biomarkers

Furthermore, linear discriminant analysis effect size (LEfSe) analysis performed across all groups revealed that the anaerobically incubated groups were characterized by multiple anaerobic taxa, including *Geomonas*, *Anaeromyxobacter*, *Neobacillus*, *Oxobacter*, and *Symbiobacteraceae* (Fig. S15) [51]. These taxa typically functioned in DIR process, extracellular hydrolysis, fermentation, and acetogenesis in anaerobic soil environments [52]. This suggested that their enrichment was consistent with the enhanced reduction and transformation of iron minerals during anaerobic incubation with A-HA. Specifically, under 180 mg/l A-HA, typical iron-reducing taxa (*Geobacteraceae*, *Geomonas*) were enriched, suggesting that this concentration provided sufficient electron shuttling capacity to directly activate DIR pathways. When the A-HA concentration increased to 300 mg/l, *Anaeromyxobacter* was additionally enriched, which is a facultative iron reducer capable of utilizing humic substances as both electron acceptors and carbon sources. This result explained why the 300 mg/l A-HA group exhibited the highest 0.5 M HCl-extracted Fe(II) concentration after 20 d of anaerobic incubation. However, at 10 d of anaerobic incubation, the 180 mg/l A-HA group displayed the highest 0.5 M HCl-extracted Fe(II) concentration. This nonmonotonic response could be attributed to the competition between the excessive A-HA input and Fe(III) minerals for electron acceptors during the early stages, which retarded the DIR process and •OH accumulation. Consequently, microbial electrons were diverted to reduce A-HA, which explained the accumulation of reducing organic matter that promoted •OH generation at 10 d of anaerobic incubation. Additionally, the high-dose A-HA input could alter the C/N, causing metabolic substrates to be preferentially utilized by organic matter decomposing bacteria, thereby limiting the development of iron-reducing bacteria. This concentration-dependent taxonomic shift indicated that higher A-HA loading not only sustained the classical DIR pathway but also recruited versatile taxa adapted to metabolizing humic substrates. Concurrently, the enrichment of *Neobacillus* in D20-300 reflected the increased demand for depolymerization and primary degradation of complex organic matter, which facilitated the provision of low molecular substrates for the DIR process. LEfSe analysis in the anaerobic groups identified no specific biomarkers for the D20-180 group (Fig. 2F). This absence suggested a concentration-dependent response of the bacterial community to A-HA, where 180 mg/l A-HA represented a transitional state with community characteristics intermediate between D20-0 and D20-300, resulting in a lack of distinct dominant taxa unique to this group. D20-0 was primarily enriched in sulfate-reducing bacteria (e.g., *Desulfocucumis* and *Desulfurisporaceae*) and oligotrophic taxa (e.g., *Patescibacteria* and *OPB41*), whereas D20-300 was enriched with hydrolytic and fermentative taxa (e.g., *Neobacillus* and *Bacillaceae*). These results further confirmed that A-HA shifted the microbial community from an oligotrophic, sulfate-reducing state to a copiotrophic state driven by organic matter decomposition and iron reduction.

In contrast, LEfSe analysis of fungal communities for all groups and anaerobic groups showed that indigenous saprophytic taxa enriched in D20-0 (e.g., *Chaetomium*, *Thermomyces*, and *Myxotrichum*) were replaced by specific fungi in A-HA-amended groups (Figs. S16 and S17). Specifically, *Rhodotorula* was identified as the indicator species for D20-180, while *Exophiala* and *Talaromyces* were significantly enriched in D20-300. This indicated that fungi played an auxiliary role in the A-HA-enhanced DIR process, primarily contributing to the decomposition of carbon skeletons to support bacterial metabolism, rather than acting as the primary drivers of iron reduction.

#### Ecological networks and environmental linkages

Furthermore, Zi-Pi analysis identified *Clostridium* and *norank_o_RBG-13-54-9* as bacterial connectors bridging distinct functional modules (Fig. S8A) [53]. These connectors occupied a critical intermediate position to maintain the DIR process: (a) primary degradation, in which hydrolytic bacteria (e.g., *Neobacillus*) and *Bacteroidota* depolymerized complex A-HA and/or native SOM into low-molecular-weight substrates; (b) intermediate fermentation, in which connectors such as *Clostridium* and *Oxobacter* fermented these substrates to produce acetate and H_2_; (c) terminal iron reduction, in which obligate and facultative iron reducers (e.g., *Geomonas* and *Anaeromyxobacter*) utilized the fermentation-derived acetate and H_2_ as preferred electron donors to reduce Fe(III) minerals. This syntrophic network ensured a continuous electron flow from complex organic carbon to Fe(III), thereby sustaining efficient DIR throughout the anaerobic incubation. In contrast, *Mrakia* functioned as a fungal module hub (Fig. S18B), indicating that fungal communities maintained a stable structure in A-HA-amended systems. Figure S19 presents the correlation heatmap between environmental factors and microbial communities, where component 1 (C1) and component 2 (C2) were derived from the parallel factor analysis (PARAFAC) modeling of DOM before oxygen exposure, and ΔDOC represented the total organic carbon difference of DOM before and after oxygen exposure. Specifically, genera belonging to hydrolytic and fermentative guilds (e.g., *Neobacillus*, *Oxobacter*, *Clostridium*, and *Fonticella*) showed significant positive correlations with Fe(II) accumulation and •OH generation (Fig. S19A). Although *Geomonas* and *Anaeromyxobacter* lacked statistical significance, the correlation trend aligned with other functional factors. In contrast, sulfate-reducing bacteria (e.g., *Desulfocucumis*) and oligotrophic indigenous taxa (e.g., *OPB41* and *Koribacter*) exhibited significant negative correlations, reflecting their competitive exclusion in A-HA system. Fungal genera like *Exophiala* and *Talaromyces* were also positively correlated with •OH and ΔDOC, validating their role in degrading A-HA into reactive precursors (Fig. S19B).

In summary, the integrated evidence from amplicon sequencing, LEfSe biomarker analysis, ecological network analysis, and environmental correlation analysis collectively indicated that A-HA reinforced the DIR process by regulating electron acceptor availability and reshaping the microbial community structure. These processes shifted the dominant functional guilds from sulfate-reducing bacteria to iron-reducing and fermentative bacteria, which was expected to accelerate iron reduction and the associated biogeochemical cycles. To further substantiate this linkage, untargeted metabolomics was subsequently employed to elucidate the metabolic mechanisms underlying the A-HA-regulated DIR process.

#### Metabolic profiles and overall differentiation

**Fig. 3.**
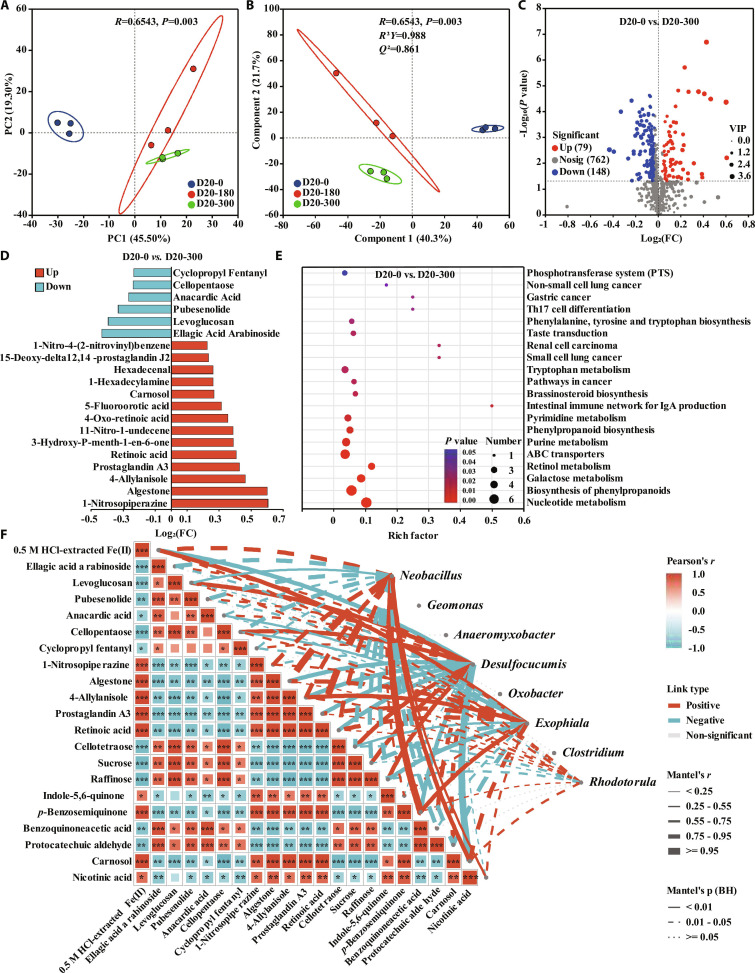
(A) PCA and (B) PLS-DA plots of metabolic profiles in D20-0, D20-180, and D20-300. (C) Volcano plot of differential metabolites between D20-0 and D20-300. (D) Fold changes of representative differential metabolites between D20-0 and D20-300. (E) KEGG pathway enrichment analysis of differential metabolites between D20-0 and D20-300. (F) Pearson correlation among environmental factors and representative metabolites, and Mantel test analysis linking microbial genera to environmental factors and metabolites.

To further elucidate the metabolic mechanisms underlying A-HA-regulated DIR processes, untargeted metabolomics was conducted for raw soil and anaerobically incubated samples (D20-0, D20-180, and D20-300). The PCA score plot of all samples revealed a clear separation between raw soil and anaerobically incubated treatments, while the quality control (QC) samples clustered closely and biological replicates grouped well (Fig. S20). These observations confirmed the high stability and reproducibility of the instrumental analysis, and indicated that anaerobic incubation reshaped the soil metabolic profiles. Furthermore, both PCA and partial least squares discriminant analysis (PLS-DA) revealed a distinct separation between A-HA-amended groups and the unamended control (Fig. 3A and B), confirming that A-HA significantly reshaped the metabolic profile of the microbial community. Additionally, the permutation test further indicated that the PLS-DA model was not overfitted (Fig. S21).

#### Differential metabolites and pathway enrichment

Differential metabolite analysis was performed to reveal the variations in metabolite abundance and composition [*P* < 0.05, variable importance in projection (VIP) > 1]. As shown in Fig. 3C, D20-300 exhibited 79 up-regulated and 148 down-regulated metabolites compared to D20-0. Specifically, several carbohydrates and phenolic compounds (ellagic acid arabinoside, levoglucosan, anacardic acid, and cellopentaose) were significantly down-regulated in D20-300 (Fig. 3D), suggesting an enhanced transformation and depolymerization of complex plant-derived precursors with high molecular weight. This phenomenon was consistent with the enrichment of hydrolytic and fermentative bacteria (e.g., *Neobacillus* and *Clostridium*), which could enhance the conversion of complex substrates into low-molecular-weight intermediates and electron donors, thereby facilitating microbial Fe(III) reduction [54]. In contrast, the metabolites enriched in D20-300 were dominated by nitrogen-containing, carboxylic acid, and lipid-like compounds (e.g., 5-fluoroorotic acid, prostaglandin A3, retinoic acid, and hexadecenal). The accumulation of these low-molecular-weight metabolites indicated intensified microbial turnover and production of readily available substrates and electron donors, which supported the enhanced DIR observed in the D20-300 treatment. It is noteworthy that the D20-300 group added a significant amount of A-HA, which contains abundant oxygen-containing functional groups (particularly quinone/semiquinone species). Therefore, the source of these substances with electron shuttle functions could consist of both abiotic transformation of A-HA and microbial synthesis. Furthermore, Kyoto Encyclopedia of Genes and Genomes (KEGG) pathway enrichment analysis (Fig. 3E) revealed that the differential metabolites were primarily enriched in nucleotide metabolism, biosynthesis of phenylpropanoids, galactose metabolism, and membrane transport systems [ATP-binding cassette (ABC) transporters and phosphotransferase system] [55]. The enrichment of nucleotide-related pathways (e.g., nucleotide, purine and pyrimidine metabolism) indicated active microbial proliferation and enhanced energy metabolism. Meanwhile, phenylpropanoids and galactose metabolism pathways provided evidence for the enhanced biotransformation of aromatic and carbohydrate compounds. Notably, the enrichment of ABC transporters and phosphotransferase systems indicated a high-flux uptake of substrates [56]. These metabolic pathways likely favored copiotrophic iron-reducing and fermentative bacteria rather than oligotrophic sulfate-reducing taxa, while the metabolic byproducts potentially functioned as electron shuttles to accelerate the DIR process [57,58].

#### Concentration-dependent metabolic characteristics

On the other hand, there were 43 up-regulated and 114 down-regulated metabolites in the comparison between D20-0 and D20-180, whereas 25 up-regulated and 39 down-regulated metabolites were identified between D20-180 and D20-300 (Fig. S22). This indicated that the introduction of A-HA was the primary driver of the metabolic shift, while the increase in concentration amplified these variations. Specifically, the metabolic alterations in D20-0 versus D20-180 mirrored the trends observed in D20-0 versus D20-300, characterized by significant down-regulation of phenolic and carbohydrate compounds and the enrichment of phenylpropanoid and galactose metabolism pathways (Fig. S23). Furthermore, the comparison between D20-180 and D20-300 revealed a down-regulation of recalcitrant carbohydrates (e.g., cellopentaose and levoglucosan) and a specific enrichment of the citrate cycle [tricarboxylic acid (TCA) cycle] (Fig. S24). These results implied that higher concentrations of A-HA stimulated more vigorous central carbon metabolism and energy production, thereby maximizing the efficiency of the DIR process. Additionally, Fig. S25 presents the top 30 differential metabolites ranked by VIP scores across the 3 anaerobic groups. D20-0 was characterized by the accumulation of carbohydrates and phenolic precursors (e.g., anacardic acid, levoglucosan, cellopentaose, sucrose, and raffinose). In contrast, D20-180 and D20-300 exhibited similar enrichment patterns but displayed a contrasting trend compared to D20-0, indicating that A-HA amendment enhanced carbon utilization efficiency. Consistently, KEGG enrichment analysis across the 3 anaerobic groups highlighted substrate transport and carbohydrate metabolism (ABC transporters, phosphotransferase system, starch and sucrose metabolism, galactose metabolism, and pentose phosphate pathway) together with phenylpropanoid biosynthesis and aromatic amino acid metabolism (tryptophan and tyrosine metabolism) (Fig. S26). Collectively, these results suggested that A-HA reshaped the metabolic pathways by accelerating carbohydrate hydrolysis and transmembrane transport, thereby promoting substrate acquisition and central carbon metabolism under anaerobic incubation. These metabolic shifts strengthened microbial energy metabolism and provided favorable metabolic conditions for the microbial iron reduction process.

#### Linkages among metabolites, microbial genera, and iron reduction

Finally, Pearson correlation analysis and Mantel test were conducted to elucidate the linkages among 0.5 M HCl-extracted Fe(II), representative differential metabolites, and key microbial genera (Fig. 3F). Pearson correlation analysis showed that 0.5 M HCl-extracted Fe(II) was significantly negatively correlated with cellulose-derived carbohydrates and phenolic precursors (e.g., ellagic acid arabinoside, levoglucosan, and cellotetraose/cellopentaose), indicating that these compounds were metabolized in the Fe(III) reduction process. In contrast, 0.5 M HCl-extracted Fe(II) exhibited significant positive correlations with quinone/semiquinone-like metabolites (*p*-benzosemiquinone, indole-5,6-quinone, benzoquinoneacetic acid, and protocatechuic aldehyde) and several metabolites enriched in D20-300 (e.g., 1-nitrosopiperazine, algestone, prostaglandin A3, retinoic acid, carnosol, and nicotinic acid). These results suggested that Fe(II) accumulation was accompanied by frequent microbial metabolism and the formation of redox-active intermediates, which could participate in Fe(III) reduction via extracellular electron transfer [57,59]. Mantel test further revealed significant statistical associations between *Neobacillus* and 0.5 M HCl-extracted Fe(II) together with multiple redox-active metabolites, particularly *p*-benzosemiquinone, retinoic acid, prostaglandin A3, algestone, and carnosol. These associations were consistent with the known functional role of *Neobacillus* in extracellular hydrolysis and primary degradation of complex organic matter, which would be expected to generate redox-active intermediates and low-molecular-weight electron donors that facilitate the DIR process. Moreover, *Exophiala* and *Rhodotorula* also showed significant associations with several metabolites, supporting the auxiliary role of fungi in carbon skeleton decomposition.

Collectively, these results provided converging evidence from multiple analytical dimensions that supported the proposed mechanistic linkage. A-HA reshaped the microbial community toward iron-reducing and fermentative functional guilds, which in turn reprogrammed metabolic pathways to accelerate the transformation of complex organic substrates into low-molecular-weight electron donors and redox-active intermediates, ultimately promoting microbial Fe(III) reduction.

### Transformation of organic matter

#### Variations in organic carbon concentration

The variations in SOM and DOM during the oxygen exposure treatment were investigated to evaluate the effect of •OH on organic matter transformation. Figures S27A and S28A to C illustrate that the concentrations of dissolved organic carbon (DOC) decreased ranging from 16.99 to 70.65 mgC/l after the oxidation treatment. Although orbital shaking for 12 h could facilitate the organic matter dissolution, the observed decrease in DOC after oxidation implicated that •OH induced DOC mineralization. Supporting this, the strong linear relationships (*R*^2^ ≥ 0.95) existed between •OH accumulation and DOC mineralization across all treatments except for 5-d anaerobic incubation, indicating that •OH was the predominant mediator for DOM mineralization (Fig. S27B). Additionally, Fe(III) generated during the Fe(II) oxidation process could contribute to the decrease in DOC through complexation and precipitation. In contrast, the concentrations of solid organic carbon (SOC) decreased ranging from 0.91 to 2.31 mgC/g soil after the oxidative treatment only in the 5-d anaerobic samples (Fig. S28D to F). The change in SOC could be jointly controlled by multiple factors, including •OH-mediated mineralization, selective adsorption and fractionation by newly formed Fe(III)(oxyhydr)oxides, and the release of microbial residues [60].

#### Variations of fluorescent components

**Fig. 4.**
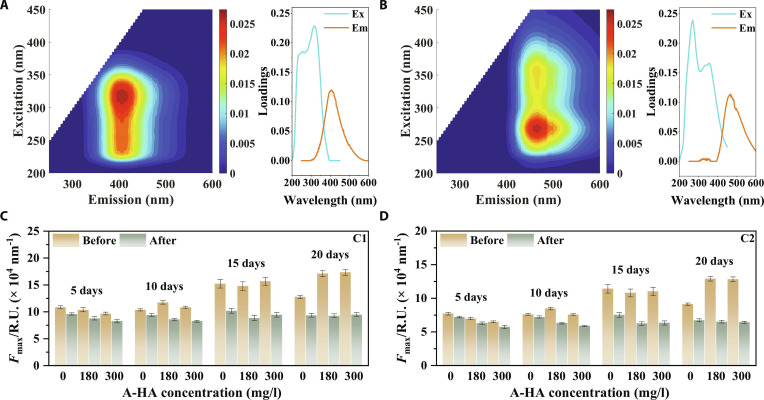
Fluorescent components and corresponding excitation/emission (Ex/Em) loadings of (A) C1 and (B) C2. *F*_max_ values for (C) C1 and (D) C2 before and after oxidation under different A-HA additions and anoxic days.

The excitation and emission (EEM) fluorescence spectra demonstrated a decrease in fluorescence intensity after the oxidative treatment compared to untreated samples (Figs. S29 to S32). The observed fluorescence decrease could be attributed to the •OH-induced mineralization, which degraded fluorescent components through oxidative decomposition. Additionally, the Fe(III)(oxyhydr)oxides formed during oxygenation could act as adsorbents. These iron minerals could preferentially adsorb and fractionate humic-like and aromatic fluorescent components via surface complexation, which also contributed to the observed decrease in EEM fluorescence intensities. Furthermore, Fig. 4A and B presents the fluorescence spectra and corresponding loadings of the 2 fluorescent components identified by the PARAFAC model constructed from all EEM spectra (Figs. S29 to S32). C1 was identified as a humic-like substance with emission/excitation (Em/Ex) located at 408/315 (245) nm [61]. C2 was characterized as a biodegradable humic-like constituent predominantly comprising hydrophobic organic matter with Em/Ex located at 468/270 (360) nm [61]. As shown in Fig. 4C and D, the *F*_max_ values for C1 and C2 before oxidation treatment ranged from 9.69 × 10^4^ to 17.33 × 10^4^ nm^−1^ and 6.50 × 10^4^ to 12.90 × 10^4^ nm^−1^, respectively. After oxidation treatment, the *F*_max_ values decreased to 8.27 × 10^4^ to 10.21 × 10^4^ nm^−1^ for C1 and 5.73 × 10^4^ to 7.53 × 10^4^ nm^−1^ for C2, suggesting that both C1 and C2 underwent degradation mediated by •OH. It is noteworthy that C2 exhibited lower *F*_max_ values than C1 across all treatments. This difference could be attributed to the hydrophobic characteristics of C2, which exhibited stronger adsorption onto solid matrices (sediments, soils, and minerals), thereby reducing its proportion within the transformation into DOM [62].

#### Molecular composition of DOM

The representative samples (D20-0-Before, D20-180-Before, and D20-180-After) were selected for molecular characterization using FT-ICR MS. The Van Krevelen diagrams displayed comparable formula distributions across these samples (Fig. S33A to E and Text S1). This observation was consistent with the fluorescence results (Fig. S32), which primarily demonstrated intensity changes rather than spectral peak shifts. These findings collectively suggested that the A-HA addition and the oxidation treatment primarily influenced the relative abundance of specific molecular components, rather than inducing transformations in the overall molecular composition. In addition, the double bond equivalent–nominal oxidation state of carbon (DBE-NOSC) plot revealed that anaerobic incubation with A-HA and oxidation treatment could alter the redox characteristics of DOM, suggesting that these alterations were reversible (Figs. S33F and S34 and Text S2).

#### Common molecular comparison of DOM

**Fig. 5.**
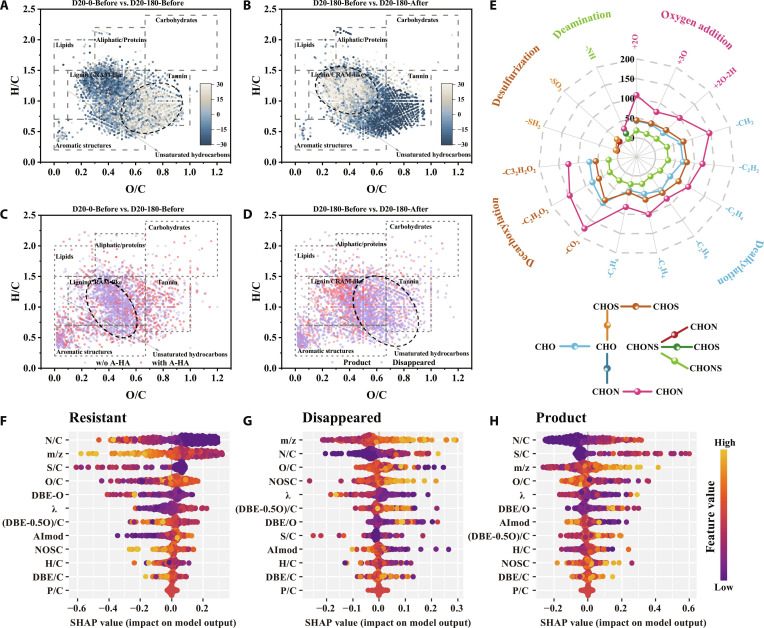
Van Krevelen diagram based on abundance variations of common molecules in (A) D20-0-Before versus D20-180-Before and (B) D20-180-Before versus D20-180-After. Van Krevelen diagram of unique molecules in (C) D20-0-Before versus D20-180-Before and (D) D20-180-Before versus D20-180-After. (E) The radar plots show the types and number of molecular reactions during the oxidation process. SHAP values of molecular properties for (F) “Resistant”, (G) “Disappeared”, and (H) “Product” comparing D20-180-Before and D20-180-After (XGBoost).

The comparative analysis was performed using D20-0-Before and D20-180-Before to investigate the effects of A-HA on DOM composition during anaerobic incubation, while comparison of D20-180-Before and D20-180-After was utilized to elucidate the •OH-driven transformation mechanisms of DOM during the oxidation process. The classification methodology, along with corresponding numbers of common and unique formulas identified in these comparisons, was described in Text S3 and Fig. S35. The abundance variations of common formulas were quantitatively analyzed using the methodology described in Text S3, and the results are presented in Fig. 5A and B. As illustrated in Fig. 5A, D20-180-Before exhibited a higher relative abundance of tannins, lignin/carboxylic-rich alicyclic molecules (CRAM)-like structures, and aromatic structures [oxygen-to-carbon atomic ratio (O/C) > 0.5] compared to D20-0-Before. The elemental analysis results indicated that A-HA had an average O/C of 0.35, suggesting that the introduction of A-HA did not alter the population of high O/C molecules (Table S1). These observations demonstrated that the anaerobic incubation in the presence of A-HA led to a relative enrichment of high O/C compounds while facilitating the decomposition of molecules with lower O/C (<0.5). This phenomenon could be attributed to the enrichment of hydrolytic and fermentative bacteria (e.g., *Neobacillus* and *Clostridium*) identified in the microbial analysis. These functional genera preferentially decomposed low O/C compounds and produced secondary molecules with high O/C through primary hydrolysis, fermentation acid production, or electron transfer to Fe(III) [63,64]. Moreover, Fig. S36A reveals that D20-180-Before exhibited an accumulation of oxidized molecules and a concomitant depletion of reduced molecules during anaerobic incubation. This finding further confirmed that the addition of A-HA promoted the utilization of reduced molecules as primary electron donors for the DIR process, subsequently driving the generation of highly oxidized secondary metabolites. Consistently, these findings provided indirect validation for the observed increase in Fe(II) content in the presence of A-HA.

In contrast, Fig. 5B reveals that D20-180-After exhibited an increased relative abundance of lignin/CRAM-like structures (O/C < 0.6) while simultaneously showing a decreased relative abundance of molecules with O/C above 0.6 compared to D20-180-Before. The results suggested that oxidative treatment preferentially decomposed high O/C compounds, leading to a relative accumulation of lower O/C compounds. This phenomenon could be attributed to the susceptibility of high O/C compounds, which contained polar functional groups (e.g., carboxyl and phenolic hydroxyl groups), to preferential attack by •OH under oxidative conditions. Concurrently, these oxygen-rich polar functional groups exhibited a high binding affinity for iron minerals, making them highly susceptible to selective adsorption and fractionation by the newly precipitated Fe(III)(oxyhydr)oxides. The observation in Fig. 4 indicated that the oxidative treatment could reduce peak intensity, providing further support for this inference. Furthermore, Fig. S36B demonstrates that formulas designated as relatively more oxidized were degraded while relatively more reduced formulas were accumulated after oxidation treatment. Taken together, the molecular transformations mediated by microbial reduction during anaerobic incubation appeared complementary to those induced by the subsequent oxidative treatment. Specifically, compounds that accumulated during anaerobic treatment preferentially tended to decompose during oxidation, supporting the reversibility of oxidation–reduction processes as demonstrated by Zhou et al. [16].

#### Unique molecular comparison of DOM

The formulas uniquely identified in the comparison between D20-0-Before and D20-180-Before are shown in Fig. 5C, and those uniquely presented in the comparison between D20-180-Before and D20-180-After are depicted in Fig. 5D. The formulas in D20-0-Before were predominantly distributed within the lignin/CRAM-like region (O/C < 0.6). In contrast, D20-180-Before exhibited a more extensive distribution in lignin/CRAM-like (O/C > 0.6) and tannin region. The distributional difference was consistent with the abovementioned intensity changes observed for formulas common to both samples (Fig. 5A). Furthermore, comparing pre- and post-oxidation samples, those formulas designated as “Disappeared” were predominantly located in the lignin/CRAM-like region (O/C > 0.4) and the majority of tannin region. In contrast, formulas labeled as “Product” were not only distributed in regions corresponding to “Disappeared” formulas but also extended into the lignin/CRAM-like region (O/C < 0.4). Collectively, these findings indicated that A-HA addition facilitated the accumulation of high O/C compounds during anaerobic incubation, while the molecular distribution in the sample without A-HA remained more concentrated in the region where O/C was less than 0.6. Furthermore, the oxidative treatment led to the preferential degradation of high O/C compounds and the generation of products (O/C < 0.4). These findings demonstrated that the redox fluctuations in the presence of A-HA drove the redistribution of molecular formulae and an increase in molecular diversity.

Furthermore, the (DBE-O)/C-NOSC analysis indicated that A-HA addition during anaerobic incubation increased both the relative abundance (+8.9%) and the absolute count of unique molecular formulas classified as “saturated and oxidized” (particularly CHO and CHON formulas), although the total counts of detectable unique formulas decreased. This phenomenon could be attributed to A-HA as a supplemental carbon source that enhanced microbial activity, thereby promoting the generation of oxidative compounds such as amino acids and low-molecular-weight organic acids. In oxygen exposure treatment, the generated •OH preferentially degraded “saturated and oxidized” compounds. The observed increase in reduced compounds reflected their resistance to •OH attack during the oxidation treatment. The detailed analysis was listed in Text S4, Figs. S37 and S38, and Tables S2 and S3.

#### Molecular transformation of DOM

The specific molecular transformation pathways during the oxidation process were identified using the paired mass distance (PMD) matching method (Text S5 and Table S4). As indicated in Fig. 4E and Table S5, dealkylation (*n* = 1,505), decarboxylation (*n* = 974), and oxygen addition (*n* = 603) represented the most significant reaction types. The most abundant elemental transformation combinations were identified as CHON-CHON (*n* = 1,392), CHO-CHO (*n* = 704), and CHOS-CHOS (*n* = 745). These findings implied that nitrogen-rich microbial metabolites accumulated during the anaerobic incubation, such as amino acids and organic acids, were likely active components in these molecular transformations. Furthermore, it was noteworthy that although CHOS molecules constituted only 5.5% to 6.4% of the molecular composition, the number of their transformations was comparable to those of CHO and CHON molecules. This finding indicated that the sulfur-containing compounds exhibited considerable sensitivity to the oxidation process. Detailed explanations for each reaction type were provided in Text S6.

#### Molecular characteristics of DOM

To elucidate the key molecular characteristics governing the DOM reactivity during the oxidation treatment, each molecule in D20-180-Before and D20-180-After was individually classified as “Disappeared”, “Resistant”, or “Product”. Concurrently, a suite of molecular characteristic parameters was calculated for each molecule (Text S7). The XGBoost, Random Forest, and LightGBM algorithms were employed to predict the association between molecular characteristics and molecular categories, all achieving prediction accuracies exceeding 76% with consistent prediction results. The SHapley Additive exPlanations (SHAP) analysis was further conducted to interpret the prediction results (Fig. 5F to H and Figs. S39 and S40), revealing that nitrogen-to-carbon atomic ratio (N/C), mass-to-charge ratio (m/z), sulfur-to-carbon atomic ratio (S/C), and O/C were key factors influencing molecular reactivity. This finding aligned with the metabolomics results, which revealed an enrichment of nucleotide and amino acid metabolism. The nitrogen-rich metabolites accumulated during the anaerobic incubation were confirmed to be preferentially degraded during the oxidative treatment. The detailed description of the interpretation was presented in Text S8.

### Degradation of contaminants

Propanil (DCPA), thiamethoxam (THM), and atrazine (ATZ) were selected as target contaminants to evaluate the removal efficiency of the oxidative treatment. As illustrated in Fig. S41, the removal rates for DCPA, THM, and ATZ within 12 h of oxygen exposure were 14.49% to 56.54%, 6.96% to 7.82%, and 4.66% to 4.72%, respectively. This finding indicated that DCPA was capable of effective degradation under redox-fluctuating conditions in paddy soil. Similar results were also reported in a study investigating contaminant degradation in rice rhizosphere iron plaque system [37]. This phenomenon was primarily attributed to the predominant generation of •OH at iron mineral interfaces. Additionally, the adsorption of DCPA onto iron mineral interfaces could facilitate its contact with •OH and subsequent degradation. Interestingly, the DCPA degradation rates with the addition of 0, 180, and 300 mg/l A-HA were 34.54%, 56.54%, and 14.49%, respectively. The degradation discrepancy could be attributed to the combined effects of •OH generation levels and organic matter quenching. The high •OH scavenging rate constant of DOM (1.0 to 3.0 × 10^4^ l mgC^−1^ s^−1^) caused excessive A-HA (300 mg/l) to outcompete DCPA, thereby decreasing removal rates [65].

**Fig. 6.**
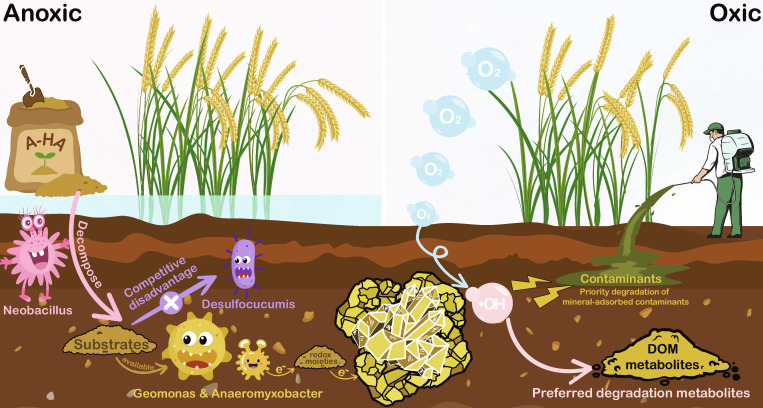
The mechanistic diagram of A-HA regulation on microbial turnover and organic matter transformation.

In summary, A-HA influenced iron reduction and •OH generation by regulating microbial communities and metabolic processes, and affected the selective transformation of organic matter during oxidation. The overall mechanism is illustrated in Fig. 6.

## Conclusion

This work provided the first insight into the influence of A-HA on the accumulation of •OH under redox fluctuation. The relationship between A-HA concentration and •OH accumulation demonstrated a close correlation with anaerobic incubation time. Further analysis indicated that A-HA primarily influenced •OH accumulation by regulating the surface Fe(II) on low-crystallinity iron minerals [measured as 0.5 M HCl-extracted Fe(II)]. Moreover, A-HA reshaped the microbial community from sulfate-reducing state to iron-reducing and fermentative state, thereby enhancing iron reduction through accelerated metabolic transformation of organic substrates. This biological process induced the enrichment of high O/C molecules and N-containing molecules during anaerobic cultivation. These molecules subsequently underwent preferential degradation through dealkylation and decarboxylation reactions during the oxidative treatment. The application of A-HA altered the dominant microbial genera and DOM transformation pathway, and accelerated the degradation of DCPA in paddy fields.

These results fundamentally shift the perception of A-HA from a passive soil conditioner to an active participant in soil biogeochemical processes under redox fluctuation. Our findings highlight the necessity for focusing on A-HA application strategies in paddy fields. Comprehensive assessments of A-HA as a soil amendment should extend beyond agronomic benefits (e.g., crop yield and soil structure) to encompass its capacity to alter redox-driven biogeochemical processes.

## Materials and Methods

### Paddy soils and chemicals

Paddy soil was collected from the surface layer (0 to 20 cm) of multi-year rice cultivation fields (47°36′28.5″N, 133°54′18.2″E) located at Qianfeng Farm, Tongjiang City, Heilongjiang Province, China. The soil sample was processed to remove straw fragments and other exogenous organic matter, followed by oven-drying at 35 °C and grinding, then sieved through a 100-mesh screen for subsequent analysis. A-HA was prepared using the method described in our previous study [22,32], with the detailed preparation procedure and parameters provided in Text S9. The chemicals employed in this study were detailed in Text S10. Unless otherwise stated, all chemicals were of analytical grade or higher purity and prepared using deionized water.

### Anaerobic incubation and oxygen exposure treatment

This study referenced the previously documented methodology of oxygen exclusion and dark incubation to simulate an anaerobic environment of paddy ecosystem under flooded conditions [7]. The anaerobically incubated suspensions were exposed to atmospheric conditions to simulate the interaction of oxygen and paddy soil. Specifically, 20 g of paddy soil was homogeneously mixed with 50 ml of either deionized water or the stock solution of A-HA (180 and 300 mg/l) in a 100-ml anaerobic vessel. The mixture was purged with nitrogen gas for 180 min, after which the vessel was hermetically sealed and incubated in the dark for 5, 10, 15, and 20 d in an orbital shaker at 25 °C. Detailed experimental methods, including the formation of •OH, sequential extraction of iron species, the extraction of organic matter, and the degradation of organic contaminants, were described in Texts S11 to S14.

### Sample analyses

The concentration of •OH was 5.87 times that of *p*-hydroxybenzoic acid (*p*-HBA), where *p*-HBA is the product from the reaction between •OH and benzoic acid (BA). It should be noted that •OH accumulation used throughout this study refers to the cumulative production of •OH, as quantified indirectly through the formation of *p*-HBA, rather than the steady-state concentration of •OH. Meanwhile, the •OH concentration calculated by this method represents the apparent accumulated concentration captured by the BA under competition kinetics, rather than the absolute total production of •OH. The concentrations of *p*-HBA, DCPA, THM, and ATZ were determined using high-performance liquid chromatography (Shimadzu, LC-20) equipped with a C18 column (5 μm, 4.6 I.D × 250 mm) and ultraviolet detector (Table S6). The concentrations of iron species were quantified utilizing the 1,10-phenanthroline colorimetric method (Text S15). The ORP of the soil suspensions was measured using a portable multi-meter (Hach, HQ4200) in an anoxic glove box. DOC and SOC were measured through total organic carbon analyzer (Shimadzu, TOC-L and SSM-5000A). The EEM fluorescence spectra were collected via a fluorescence spectrometer (Shimadzu, RF-6000). PARAFAC was employed to classify the characteristic peaks of EEM and quantify the corresponding weights. FT-ICR MS (Bruker, SolariX-15T) was employed to determine mass-to-charge ratio (m/z), intensities, and signal-to-noise ratios of DOM, with detailed detection mass parameters listed in Text S16. The solid-phase extraction protocols were detailed in Text S17, and the molecular formula assignment was performed according to the method reported by Goranov et al. [66] (details in Text S18). The eXtreme gradient boosting algorithm (XGBoost) was employed to identify descriptors sensitive to oxidation reactions, while SHAP analysis was implemented to interpret and visualize the machine learning results (Text S19) [13,67]. The class assignment and characteristic parameters of formulas were detailed in Text S7.

### Microbial community sequencing and analysis

Soil DNA was isolated from soil samples utilizing the E.Z.N.A. Soil DNA Kit (Omega Bio-tek, USA). The bacterial 16S rRNA gene (V3–V4) and fungal ITS region were amplified using primers 338F/806R and ITS1F/ITS2R, respectively. Sequencing was conducted on an Illumina NextSeq 2000 platform at Majorbio Bio-Pharm Technology Co. Ltd. (Shanghai, China). Raw reads were quality-filtered by fastp (v0.19.6) and merged using FLASH (v1.2.7). The DADA2 algorithm was employed to denoise sequences and generate amplicon sequence variants (ASVs). Taxonomic assignment was performed using the classify-sklearn (Naive Bayes) estimator with a confidence threshold of 0.7. Bacterial and fungal ASVs were annotated against the SILVA (v138.2) and UNITE (v9.0) databases, respectively. All diversity analyses were executed on the Majorbio Cloud Platform (https://www.majorbio.com).

### Nontargeted metabolomics analysis

Nontargeted metabolomic profiling of the soil samples was carried out using a UHPLC-Q Exactive 480 mass spectrometer (Thermo Fisher Scientific, USA) at Majorbio Bio-Pharm Technology Co. Ltd. (Shanghai, China). Briefly, metabolites were extracted from the soil using a methanol:water mixture (4:1, v/v) spiked with an internal standard (L-2-chlorophenylalanine). The mixture was homogenized, sonicated at low temperature, and centrifuged to recover the supernatant for subsequent liquid chromatography–tandem mass spectrometry (LC-MS/MS) analysis. QC samples, prepared by pooling equal aliquots of all experimental samples, were inserted regularly throughout the run to monitor instrument stability and reproducibility. All raw data processing and statistical analyses were executed via the Majorbio Cloud Platform (https://cloud.majorbio.com).

## Data Availability

The data are freely available upon request.

## Supplementary Materials

Supplementary 1 Texts S1 to S19 Figs. S1 to S41 Tables S1 to S6 References [68–80]
