# Supplementary material for: Integration of Microbial Metabolic Regulation and Abiotic Oxidation: Mechanisms of Artificial Humic Acid-Enhanced Hydroxyl Radical Generation in Paddy Soil
Source: Research (Wash D C). 2026 May 7;9:1269. doi: 10.34133/research.1269 (PMC13150079; doi:10.34133/research.1269)
Supplement: Supplementary 1 — Texts S1 to S19 Figs. S1 to S41 Tables S1 to S6 References [68–80] [file research.1269.f1.docx]

**SUPPLEMENTARY MATERIALS**

**for**

**Integration of Microbial Metabolic Regulation and Abiotic Oxidation: Mechanisms of Artificial Humic Acid-Enhanced Hydroxyl Radical Generation in Paddy Soil**

Shishun Wang^1,2^, Taiping Zhang^1,2^, Shuang Gai^1,2^, Fan Yang^1,2*^, Kui Cheng^2,3*^, Zhuqing Liu^2,3*^

^1^ School of Water Conservancy and Civil Engineering, Northeast Agricultural University, 150030, Harbin, China

^2^ International Cooperation Joint Laboratory of Health in Cold Region Black Soil Habitat of the Ministry of Education, 150030, Harbin, China

^3^ College of Engineering, Northeast Agricultural University, 150030, Harbin, China

*Corresponding author:

Fan Yang: [yangfan_neau@163.com](mailto:yangfan_neau@163.com)

Kui Cheng: [chengkui@neau.edu.cn](mailto:chengkui@neau.edu.cn)

Zhuqing Liu: [lzq@cau.edu.cn](mailto:lzq@cau.edu.cn)

**Contents Page**

[Part S1 Supplementary Texts 1](#_Toc224847544)

[**Text S1.** The analysis of the Van Krevelen diagram for D20-0-Before, D20-180-Before and D20-180-After. 1](#_Toc224847545)

[**Text S2.** The analysis of the plot of DBE-NOSC. 2](#_Toc224847546)

[**Text S3.** The calculation method for changes in common molecular intensity. 3](#_Toc224847547)

[**Text S4.** The analysis of the (DBE-O)/C-NOSC plot. 4](#_Toc224847548)

[**Text S5.** The paired mass distance (PMD) matching method. 7](#_Toc224847549)

[**Text S6.** Detailed explanations for each reaction type. 8](#_Toc224847550)

[**Text S7.** The class assignment and characteristic parameters of formulas. 9](#_Toc224847551)

[**Text S8.** The relationship between the molecular reactivity and molecular characteristics. 11](#_Toc224847552)

[**Text S9.** The preparation of A-HA and the formulation of stock solutions. 12](#_Toc224847553)

[**Text S10.** The chemicals employed in this study. 13](#_Toc224847554)

[**Text S11.** The formation of •OH. 14](#_Toc224847555)

[**Text S12.** The sequential extraction of iron species. 15](#_Toc224847556)

[**Text S13.** The extraction of organic matter. 16](#_Toc224847557)

[**Text S14.** The degradation of organic contaminants. 17](#_Toc224847558)

[**Text S15.** The detailed measurement procedures of Fe(II) species. 18](#_Toc224847559)

[**Text S16.** The detection parameters of FT-ICR MS. 19](#_Toc224847560)

[**Text S17.** The solid-phase extraction protocols. 20](#_Toc224847561)

[**Text S18.** The molecular formula assignment parameters. 21](#_Toc224847562)

[**Text S19.** Machine learning and SHAP analysis 22](#_Toc224847563)

[Part S2 Supplementary Figure 23](#_Toc224847564)

[**Fig. S1.** (a) The concentration of accumulated •OH upon oxygen exposure following 20 days of oxic incubation. (b) The concentration of accumulated •OH upon nitrogen exposure following 20 days of anoxic incubation. 23](#_Toc224847565)

[**Fig. S2.** The concentration of accumulated •OH at different oxygen exposure times following anaerobic incubation for (a) 5 days, (b) 10 days, (c) 15 days and (d) 20 days. 24](#_Toc224847566)

[**Fig. S3.** The concentration of dissolved Fe(II) at different oxygen exposure times following anaerobic incubation for (a) 5 days, (b) 10 days, (c) 15 days and (d) 20 days. 25](#_Toc224847567)

[**Fig. S4.** The concentration of 0.5 M HCl-extracted Fe(II) at different oxygen exposure times following anaerobic incubation for (a) 5 days, (b) 10 days, (c) 15 days and (d) 20 days. 26](#_Toc224847568)

[**Fig. S5.** (a) The initial ORP value at different anaerobic incubation times. (b) The correlation between the •OH accumulated and the ORP value. (c) The correlation between the initial 0.5 M HCl-extracted Fe(II) and the ORP value. 27](#_Toc224847569)

[**Fig. S6.** (a) The effect of scavengers on accumulated •OH after 20 days of anaerobic incubation. The effect of (b) BPY and (c) CAT on the concentration of accumulated •OH at different oxygen exposure times after 20 days of anaerobic incubation. 28](#_Toc224847570)

[**Fig. S7.** The rarefaction curves of (a) bacterial and (b) fungal communities. 29](#_Toc224847571)

[**Fig. S8.** The Chao index of (a) bacterial and (b) fungal communities. 30](#_Toc224847572)

[**Fig. S9.** The PCA of (a) bacterial and (b) fungal communities in D20-0, D20-180, D20-300 and Raw soil. (c) The PCA of fungal communities in D20-0, D20-180 and D20-300. 31](#_Toc224847573)

[**Fig. S10.** The βNTI of (a) bacterial and (b) fungal communities in D20-0, D20-180, D20-300 and Raw soil. 32](#_Toc224847574)

[**Fig. S11.** The Venn diagram of (a) bacterial and (b) fungal communities and the relative abundance of (c) bacterial and (d) fungal communities at the phylum level. 33](#_Toc224847575)

[**Fig. S12.** The Venn diagram of (a) bacterial and (b) fungal communities and the relative abundance of (c) bacterial and (d) fungal communities at the genus level. 34](#_Toc224847576)

[**Fig. S13.** The phylum-level differential abundance comparison of bacterial communities among D20-0, D20-180 and D20-300 by Kruskal-Wallis H test. 35](#_Toc224847577)

[**Fig. S14.** The genus-level differential abundance comparison of fungal communities among D20-0, D20-180 and D20-300 by Kruskal-Wallis H test. 36](#_Toc224847578)

[**Fig. S15.** The LDA score plot of LEfSe analysis of bacterial communities among D20-0, D20-180, D20-300 and Raw soil. 37](#_Toc224847579)

[**Fig. S17.** The LDA score plot of LEfSe analysis of fungal communities among D20-0, D20-180 and D20-300. 39](#_Toc224847580)

[**Fig. S18.** The Zi-Pi plot of (a) bacterial and (b) fungal ecological networks in D20-0, D20-180 and D20-300. 40](#_Toc224847581)

[**Fig. S19.** The correlation heatmap between environmental factors and (a) bacterial and (b) fungal communities at the genus level. 41](#_Toc224847582)

[**Fig. S20.** The PCA plot of metabolic profiles in raw soil and anaerobically incubated samples (D20-0, D20-180 and D20-300) with quality control (QC) samples. 42](#_Toc224847583)

[**Fig. S21.** The permutation test of the PLS-DA model for metabolic profiles in D20-0, D20-180 and D20-300. 43](#_Toc224847584)

[**Fig. S22.** The volcano plots of differential metabolites between (a) D20-0 and D20-180 and (b) D20-180 and D20-300. 44](#_Toc224847585)

[**Fig. S23.** (a) The fold changes of representative differential metabolites between D20-0 and D20-180. (b) The KEGG pathway enrichment analysis of differential metabolites in comparisons of D20-0 vs. D20-180. 45](#_Toc224847586)

[**Fig. S24.** (a) The fold changes of representative differential metabolites between D20-180 and D20-300. (b) The KEGG pathway enrichment analysis of differential metabolites in comparisons of D20-180 vs. D20-300. 46](#_Toc224847587)

[**Fig. S25.** The VIP scores of differential metabolites identified by PLS-DA across D20-0, D20-180, and D20-300. 47](#_Toc224847588)

[**Fig. S26.** The KEGG pathway enrichment analysis of differential metabolites identified across D20-0, D20-180, and D20-300. 48](#_Toc224847589)

[**Fig. S27.** (a) The DOC variation before and after oxygen exposure. (b) The correlation between the accumulated of •OH and the variation of DOC. 49](#_Toc224847590)

[**Fig. S28.** The concentrations of DOC before and after oxidation under conditions of (a) without A-HA, (b) with 180 mg/L A-HA and (c) with 300 mg/L A-HA. The concentration of SOC before and after oxidation under conditions of (d) without A-HA, (e) with 180 mg/L A-HA and (f) with 300 mg/L A-HA. 50](#_Toc224847591)

[**Fig. S29.** The EEM fluorescence spectra of DOM extracted from soil suspension with the addition of 0 ((a) and (d)), 180 ((b) and (e)) and 300 ((c) and (f)) mg/L A-HA before and after oxygen exposure treatment following anaerobic incubation for 5 days. 51](#_Toc224847592)

[**Fig. S30.** The EEM fluorescence spectra of DOM extracted from soil suspension with the addition of 0 ((a) and (d)), 180 ((b) and (e)) and 300 ((c) and (f)) mg/L A-HA before and after oxygen exposure treatment following anaerobic incubation for 10 days. 52](#_Toc224847593)

[**Fig. S31.** The EEM fluorescence spectra of DOM extracted from soil suspension with the addition of 0 ((a) and (d)), 180 ((b) and (e)) and 300 ((c) and (f)) mg/L A-HA before and after oxygen exposure treatment following anaerobic incubation for 15 days. 53](#_Toc224847594)

[**Fig. S32.** The EEM fluorescence spectra of DOM extracted from soil suspension with the addition of 0 ((a) and (d)), 180 ((b) and (e)) and 300 ((c) and (f)) mg/L A-HA before and after oxygen exposure treatment following anaerobic incubation for 20 days. 54](#_Toc224847595)

[**Fig. S33.** The Van Krevelen diagrams of (a) D20-0-Before, (b) D20-180-Before and (c) D20-180-After. The relative abundance of different samples according to (d) compound classes and (e) element classes. (f) The NOSC-DBE plots of different samples. 55](#_Toc224847596)

[**Fig. S34.** The distribution of formula counts in the value of DBE and NOSC (a, c) for samples D20-0-Before and D20-180-Before. The distribution of formula counts in the value of DBE and NOSC (b, d) for samples D20-180-Before and D20-180-After. 56](#_Toc224847597)

[**Fig. S35.** (a) The number and proportion of unique and common molecular formulas in the comparison between the D20-0-Before and D20-180-Before. (b) The number and proportion of disappeared, product and resistant molecular formulas in the comparison between the D20-0-Before and D20-180-Before. 57](#_Toc224847598)

[**Fig. S36.** (a) The (DBE-O/C)-NOSC plot of molecular formulas commonly present in D20-0-Before and D20-180-Before with color-coded based on the intensity difference. (b) The (DBE-O/C)-NOSC plot of molecular formulas commonly present in D20-180-Before and D20-180-After with color-coded based on the intensity difference. 58](#_Toc224847599)

[**Fig. S37.** The (DBE-O/C)-NOSC plot of molecular formulas uniquely present in (a) D20-0-Before and (b) D20-180-Before. (c) The counts percentage across different categories of molecular formulas uniquely present in D20-0-Before and D20-180-Before. 59](#_Toc224847600)

[**Fig. S38.** The (DBE-O/C)-NOSC plot of molecular formulas uniquely present in (a) D20-180-Before and (b) D20-180-After. (c) The counts percentage across different categories of molecular formulas uniquely present in D20-180-Before and D20-180-After. 60](#_Toc224847601)

[**Fig. S39.** The SHAP value of “Disappeared”, “Resistant”, and “Product” across various characteristic parameters. 61](#_Toc224847602)

[**Fig. S40.** The SHAP values of molecular properties calculated by (a) Random Forest and (b) LightGBM. 62](#_Toc224847603)

[**Fig. S41.** The degradation trends of (a) DCPA, (b) THM and (c) ATZ at different oxygen exposure times after 20 days of anaerobic incubation. 63](#_Toc224847604)

[Part S3 Supplementary Tables 64](#_Toc224847605)

[**Table S1.** The elemental composition of A-HA. 64](#_Toc224847606)

[**Table S2.** The formula counts of D20-0-Before and D20-180-Before in various element and species category. 65](#_Toc224847607)

[**Table S3.** The formula counts of D20-180-Before and D20-180-After in various element and species category. 66](#_Toc224847608)

[**Table S4.** The list of paired mass distance. 67](#_Toc224847609)

[**Table S5.** The list of molecular reaction count. 68](#_Toc224847610)

[**Table S6.** The detailed analytical parameters of p-HBA, DCPA, THM and ATZ. 69](#_Toc224847611)

**Part S1 Supplementary Texts**

**Text S1.** The analysis of the Van Krevelen diagram for D20-0-Before, D20-180-Before and D20-180-After.

The Van Krevelen diagram was divided into seven distinct regions (Text S7). Each molecular species was located within the diagram based on O/C and H/C ratios, with color-coded representations indicating different atomic compositions. The three samples exhibited comparable distribution (Fig. S33 (a-d)) with lignin/carboxylic-rich alicyclic molecules like (CRAM-like) (68.6% - 70.3%), tannin (13.1% - 14.9%), and aromatic structures (11.3% - 12.4%) emerging as the predominant molecular constituents. In addition, Fig. S33 (e) demonstrated that the elemental composition was predominantly comprised of CHON, CHO, and CHONS, with their respective proportions accounting for 45.9% - 49.7%, 34.2% - 35.7%, and 10.4% - 12.9% of the total composition. These results demonstrated that neither the addition of A-HA nor oxidation treatment could induce significant alterations in either the molecular species composition or elemental composition of DOM.

**Text S2.** The analysis of the plot of DBE-NOSC.

The elemental composition of molecules could influence the redox properties of DOM. Therefore, the normal oxidation state of carbon (NOSC) and double bond equivalence (DBE) were calculated for each molecule in the three samples and presented in Fig. S33 (f). The results demonstrated that D20-180-Before exhibited a broader distribution in NOSC and DBE plot compared to the other two samples, indicating that the addition of A-HA altered the overall redox characteristics. Specifically, Fig. S34 (a) revealed that D20-0-Before contained a greater number of molecular formulas within the DBE range of 10-15 compared to D20-180-Before, suggesting that A-HA addition modified the redox characteristics of DOM during anaerobic incubation. This phenomenon could be attributed to the enhanced microbial degradation of complex high-DBE substrates, driven by the enrichment of hydrolytic and fermentative taxa (e.g., *Neobacillus*). Interestingly, it was observed in Fig. S34 (b) that the number of molecules within the DBE range of 10-15 for D20-180-After increased to levels comparable to D20-0-Before. This observation suggested that oxidative treatment generated substances with higher degrees of unsaturation. These findings were consistent with previous research, which demonstrated the reversibility of changes occurring in NOM during reduction and oxidation processes [16, 68-70]. Similarly, the observed NOSC variations exhibited analogous patterns to variations of DBE (Fig. S34 (c-d)), suggesting that anaerobic incubation and oxidation treatment could not alter the molecular classification of DOM, but could modify the redox characteristics of specific molecular species.

**Text S3.** The calculation method for changes in common molecular intensity.

As depicted in Fig. S35 (a), the number of formulas unique to D20-0-Before and D20-180-Before were 1449 (17.8%) and 1012 (12.4%), respectively, while the number of molecules common to both samples was 5677 (69.8%). On the other hand, the molecules detected in D20-180-After were the products formed by the reaction of molecules present in D20-180-Before with •OH. Therefore, molecules presented only in D20-180-Before were classified as “Disappeared”, those present only in D20-180-After were classified as “Product”, and those common to both samples were classified as “Resistant”. This categorization was depicted in Fig. S35 (b) and the number of formulas for “Disappeared”, “Resistant”, and “Product” were 1125 (13.7%), 5564 (67.5%), and 1545 (18.8%), respectively.

Firstly, the relative magnitude of each molecule (*i*) is calculated according to Eq. S1. Subsequently, the change (Change *_i_*) for a given molecule is computed by subtracting its relative magnitude in the initial sample from its relative magnitude in the altered sample, and then normalizing this result with respect to the relative magnitude in the initial sample (Eq. S2).

**$Rel.Magnitude_{i}=\frac{Magnitude_{i}}{\sum_{1}^{n} Magnitude_{i}}$ (**Eq. S1)

**$Change_{i}=\frac{Rel.Magnitude (Sample 2)_{i}-Rel.Magnitude (Sample 1)_{i}}{Rel.Magnitude (Sample 1)_{i}}$ (**Eq. S2)

**Text S4.** The analysis of the (DBE-O)/C-NOSC plot.

Double bond equivalent minus oxygen per carbon ((DBE-O)/C) and NOSC serve to indicate the degree of unsaturation and the oxidation state of molecules, respectively. Accordingly, the (DBE-O)/C-NOSC plot enables the classification of sample components into four distinct categories: “unsaturated and oxidized”, “unsaturated and reduced”, “saturated and reduced”, and “saturated and oxidized”. In the comparison between D20-0-Before and D20-180-Before, the distributions of uniquely identified molecular formulas within the (DBE-O)/C-NOSC plot classified by elemental composition were displayed in Fig. S37 (a-b), respectively. The corresponding categories proportions and molecular counts were listed in Fig. S37 (c) and Table S2. The results showed that the proportion of unique molecules classified as “saturated and oxidized” was 8.9% higher in the sample with A-HA addition relative to the sample without A-HA. Conversely, the proportions for the other three categories were lower in the sample of A-HA addition. This finding suggested that despite a reduction in the total count of unique molecules upon A-HA addition (Table S2), the addition of A-HA facilitated the generation of a greater number of molecules characterized as “saturated and oxidized” during anaerobic incubation. This outcome aligned with results derived from Van Krevelen diagrams for both unique and common molecules, providing further support for the conclusion that A-HA addition induced the formation of substances with high oxidation states.

Furthermore, it was recognized that microbial decomposition of complex substrates often generated a range of small-molecule organic acids and nitrogenous metabolites [71-74]. This aligns with the metabolomics results, which revealed significant upregulation of nucleotide and amino acid metabolism pathways. These common microbial metabolites (e.g., 5-fluoroorotic acid, amino acid derivatives) characteristically possess saturated structures and are highly oxidized. Specifically, the absolute counts of unique CHON molecules within the “saturated and oxidized” category increased (Table S2). This observation strongly supports the hypothesis that the A-HA-reshaped microbial community actively synthesized these nitrogen-rich metabolic intermediates, which subsequently served as precursors for •OH-mediated transformation.

On the other hand, Fig. S38 (a) presented the distribution of unique formulas in D20-180-Before within the (DBE-O)/C-NOSC diagram based on elemental composition, while Fig. 38 (b) displayed the corresponding distribution for D20-180-After. The corresponding molecular counts and category proportions were detailed in Table S3 and Fig. S38 (c). In comparison to D20-180-Before, D20-180-After exhibited a 10.0% decrease in “saturated and oxidized” and a 5.3% decrease in “unsaturated and oxidized”, while demonstrating increases of 4.1% and 11.2% in “saturated and reduced” and “unsaturated and reduced”, respectively. These results suggested that the oxidation treatment preferentially degraded or transformed molecules with oxidized characteristics that were enriched in anaerobic phase. These transformed or degraded molecules likely correspond to relatively labile microbial metabolites (e.g., organic acids), which were susceptible to decarboxylation initiated by hydrogen abstraction or addition reactions with •OH. Furthermore, it could be seen from Table S3 that the decline in the proportions of “unsaturated and oxidized” and “saturated and oxidized” were primarily attributable to a reduction in the number of CHO molecules (decreasing from 121 to 24 for “saturated and oxidized”, and from 96 to 54 for “unsaturated and oxidized”). This indicated that oxidized components containing only CHO were the predominant drivers of DOM transformation induced by •OH. In contrast, the increased proportion of “unsaturated and reduced” resulted from an increase in the number of molecules across all elemental types, confirming the inertness of these reduced molecules during oxidation treatment. Additionally, the increase in “saturated and reduced” was principally driven by elevated counts of CHON (from 43 to 79) and CHOS (from 22 to 103) molecules, suggesting that molecules from the common molecular pool could participate in reactions and transform into “Product”.

**Text S5.** The paired mass distance (PMD) matching method.

The transformation of DOM induced by •OH typically involved the gain or loss of characteristic functional groups, such as the increase in hydroxyl moieties resulting from •OH addition. Consequently, specific molecular transformation pathways between samples could be identified by the paired mass distance (PMD), which represented the precise mass difference between two molecules. To elucidate the specific molecular transformation pathways arising from the interaction between •OH and DOM during the oxidation treatment, a list of typical PMD (Table S4) was established based on previously reported mechanisms of •OH-induced DOM transformation [75-78]. This list encompassed key reaction types, including oxygen addition (+2O, +3O, +2O-2H), dealkylation (-C_x_H_y_), decarboxylation (-C_x_H_y_O_z_), desulfurization (-SH_2_, -SO_3_), and deamination (-NH). The transformation pathways for specific molecular pairs were determined by comparing the precise mass differences between each molecule detected in D20-180-After and all molecules detected in D20-180-Before against the established PMD list in Table S4.

**Text S6.** Detailed explanations for each reaction type.

It could be found in Table S5 that dealkylation reactions (n=1505) primarily involved the loss of alkyl or unsaturated hydrocarbon fragments (e.g., -CH_2_, -C_2_H_2_, -C_2_H_4_, -C_2_H_6_, -C_3_H_4_, -C_3_H_6_) from molecules [79], this process has been commonly detected across molecular transformations involving diverse elemental compositions. Furthermore, decarboxylation reactions (n=974, involving the loss of -CO_2_, -C_2_H_2_O_2_, and -C_3_H_2_O_2_) predominantly occurred within the molecular composition categories of CHO-CHO (n=247) and CHON-CHON (n=448). This observation suggested that decarboxylation reactions were primarily driven by microbial metabolites and small organic acids enriched during the anaerobic phase. Additionally, the reduction in groups such as -CO_2_, -C_2_H_2_O_2_, and -C_3_H_2_O_2_ was typically associated with a decrease in NOSC. This indicated that the observed reduction in oxidative substances during the aerobic treatment phase could be attributed to decarboxylation reactions. Moreover, oxygen addition reactions (including +2O, +3O, +2O-2H) were frequently observed in the transformations of CHO and CHON molecules. This finding indicated that •OH not only facilitated DOM degradation but could also directly add to unsaturated bonds, aromatic rings, or oxygen-containing functional groups. Additionally, the transformation of heteroatom-containing molecules has been detected, indicating that •OH could attack C-S and C-N bonds, leading to the loss of heteroatoms.

**Text S7.** The class assignment and characteristic parameters of formulas.

*The class assignment of formulas.* The assigned formulae were categorized to seven regions based on O/C and H/C ratios as follows: lipids (H/C=1.5-2.0, O/C=0-0.3), aliphatic/proteins (H/C=1.5-2.2, O/C=0.3-0.67), lignin/CRAM-like (H/C=0.7-1.5, O/C=0.1-0.67), carbohydrates (H/C=1.5-2.4, O/C=0.67-1.2), unsaturated hydrocarbons (H/C=0.7-1.5, O/C=0-0.1), aromatic structures (H/C=0.2-0.7, O/C=0-0.67), and tannins (H/C=0.6-1.5, O/C=0.67-1.0).

*The characteristic parameters of formulas.* m/z is mass-to-charge ratio defined as the measured value with accuracy of 1 ppm. The method for calculating λ was adapted from procedures outlined in previous research [80]. The remaining characteristic parameters were calculated according to Eq. S3 – S12.

$\text{O/C=}\frac{\text{Number of O}}{\text{Number of C}}$ Eq. S3

$\text{H/C=}\frac{\text{Number of H}}{\text{Number of C}}$ Eq. S4

$\text{N/C=}\frac{\text{Number of N}}{\text{Number of C}}$ Eq. S5

$\text{S/C=}\frac{\text{Number of S}}{\text{Number of C}}$ Eq. S6

$\text{P/C=}\frac{\text{Number of P}}{\text{Number of C}}$ Eq. S7

$\text{AI}_{\text{mod}}\text{=}\frac{\text{1+C-}\frac{\text{O}}{2}\text{-S-}\frac{\text{N+P+H}}{2}}{\text{C-}\frac{\text{O}}{2}\text{-N-S-P}}$ Eq. S8

$\text{NOSC=4-}\frac{\text{4C+H-3N-2O-2S+5P}}{\text{C}}$ Eq. S9

$\text{DBE-O=1+}\frac{\text{2C-H+N+P}}{2}\text{-O}$ Eq. S10

$\text{DBE/C=(1+}\frac{\text{2C-H+N+P}}{2}\text{)/C}$ Eq. S11

$\text{(DBE-0.5O)/C=(1+}\frac{\text{2C-H+N+P}}{2}\text{-0.5O)/C}$ Eq. S12

**Text S8.** The relationship between the molecular reactivity and molecular characteristics.

Fig. 5 (F) demonstrated that N/C, m/z, and S/C were negatively correlated with the prediction of “Resistant”, whereas O/C was positively correlated. This indicated that molecules characterized by lower N/C, m/z, S/C, or higher O/C tended to be preferentially retained during oxidation treatment. Additionally, Fig. 5 (G) indicated that m/z, N/C, and NOSC positively contribute to the prediction of “Disappeared”, suggesting that molecules with higher m/z, N/C, and NOSC were more susceptible to degradation. This observation provided molecular-level evidence corroborating the metabolomic findings, confirming that nitrogen-containing microbial metabolites (products of upregulated nucleotide/amino acid metabolism) accumulated during anaerobic cultivation tended to be preferentially degraded during oxidation treatment, and that sulfur-containing molecules were sensitive during the oxidation process. Furthermore, N/C, S/C, and m/z were found to be positively correlated with the prediction of “Product” (Fig. 5 (H)). The conclusions drawn from this were broadly consistent with the prediction outcomes for “Resistant” and “Disappeared”. However, it was noteworthy that while S/C displayed opposing correlations concerning the “Resistant” and “Product” predictions, it possessed substantial absolute SHAP values for both categories, contrasting with its lower sensitivity towards “Disappeared”. This result suggested that sulfur-containing molecules identified within the “Product” pool likely originated from the transformation of “Resistant” rather than from “Disappeared”.

**Text S9.** The preparation of A-HA and the formulation of stock solutions.

A-HA was synthesized following our previously reported procedure [22]. Briefly, a mixture of lignin and cellulose (1.20 g, 7:3 w/w) and varying masses of KOH were placed in a 50 mL autoclave. The sealed vessel was heated at 200℃ for 24 h. After cooling to room temperature, the reaction mixture was acidified to pH 3 using 6.0 M HCl. The resulting precipitate was collected by filtration, washed, dried, and ground. The stock solutions were prepared by dissolving the solid in NaOH solution (pH 11). Following complete dissolution, the solutions were neutralized to pH 7 with HCl. The stock solutions were stored in brown serum bottles at 4℃ under oxygen exclusion and prepared fresh weekly.

**Text S10.** The chemicals employed in this study.

Benzoic acid (BA, 99%), p-Hydroxybenzoic acid (*p*-HBA, 99%), hydroxylamine hydrochloride (NH_2_OH·HCl, 98%), sodium hydroxide (NaOH, 99%) and 1,10-phenanthroline were procured from Sinopharm Chemical Reagent Co., Ltd. Ammonium acetate (CH_3_COONH_4_, 99%), acetic acid (CH_3_COOH, 99%), sodium diphosphate (Na_4_P_2_O_7_, 99%) and sodium hydrosulfite (Na_2_S_2_O_4_, 98%) were obtained from Shanghai RHAWN Chemical Technology Co., Ltd. 2,2’-dipyridyl (BPY, 98%), catalase (CAT, 98%), nitro tetrazolium blue chloride (NBT, 98%), thiamethoxam (THM, 99%), atrazine (ATZ, 99%), and propanil (DCPA, 99%) were purchased from Shanghai Macklin Biochemical Corporation. Methanol (CH_3_OH, HPLC) was obtained from TEDIA Co. Ltd.

**Text S11.** The formation of •OH.

10 mL of incubated soil suspension was combined with 10 mL of 20 mM BA in an anaerobic glove box [7]. Under the principle of competition kinetics, this concentration of BA effectively eliminates the measurement errors caused by the competitive scavenging of •OH by DOM. The mixture was subsequently exposed to air and shaken on an orbital shaker at 150 rpm for 12 h. The soil suspensions treated with 180 mg/L A-HA and anaerobically incubated for 20 days were designated as D20-180-Before and D20-180-After depended on oxygen exposure status, with this nomenclature being consistently applied to the other samples. At predetermined time intervals, 1 mL of mixture was withdrawn and mixed with 1 mL of methanol to quench the reaction and extract p-HBA (BA + •OH → p-HBA). The extract was centrifuged at 12,000 rpm for 8 min and filtered through a 0.22 μm filter membrane prior to the detection of accumulated •OH. Particularly, 20 mM BPY and 1,000 U/mL CAT were co-introduced with BA into the mixture to quench iron species and H_2_O_2_ during oxygen exposure treatment, respectively.

**Text S12.** The sequential extraction of iron species.

10 mL of incubated soil suspension was combined with 10 mL of deionized water in an anaerobic glove box. The mixture was subsequently exposed to atmosphere and shaken on an orbital shaker at 150 rpm for 8 h. At predetermined time intervals, 2 mL mixture was collected and centrifuged immediately at 12,000 rpm for 5 min. The supernatant was preserved in 1 M HCl for the determination of dissolved Fe(II). The remaining soil was treated with 2 mL of 0.5 M HCl and extracted on an orbital shaker for 2 h, followed by centrifugation at 12,000 rpm for 5 min. The supernatant was preserved in 1 M HCl at 4℃ for the quantification of surface-complexed Fe(II) and low-crystalline Fe(II).

**Text S13.** The extraction of organic matter.

The anoxic suspension after incubation was transferred to centrifuge tubes within an anaerobic glovebox and centrifuged at 8000 rpm for 5 min. Subsequently, the supernatant was filtered through a 0.45 μm filter membrane under nitrogen atmosphere and stored at 4℃. The remaining soil was immediately transferred to an ultra-low temperature freezer (-80℃) and preserved under nitrogen atmosphere for subsequent analysis after vacuum freeze-drying. The suspension after incubation was exposed to air and shaken on an orbital shaker at 150 rpm for 12 h to obtain the oxidized organic matter. The oxidized organic matter was extracted and stored using the same method as that employed for the extraction and preservation of the pre-oxidized organic matter.

**Text S14.** The degradation of organic contaminants.

DCPA, THM, and ATZ were selected as target contaminants to investigate the removal efficiency during the oxidation process. DCPA, THM and ATZ were introduced into soil suspensions incubated anaerobically for 20 days, which were subsequently removed from the glove box to contact with oxygen and start oxygen exposure treatment. At predetermined time intervals, 1 mL mixture was collected and combined with 1 mL methanol to terminate the oxidation reaction and extract organic contaminants. The samples were extracted by shaking for 24 h and centrifuged, then filtered through a 0.22 μm filter membrane and preserved at -4°C.

**Text S15.** The detailed measurement procedures of Fe(II) species.

The concentration of Fe(II) was determined using a colorimetric method with 1,10-phenanthroline. Briefly, 1 mL of supernatant was mixed with 1.5 mL of 1,10-phenanthroline monohydrate solution (0.2 g/L). The mixture was allowed to react for 30 minutes to ensure complete complexation of Fe(II) with 1,10-phenanthroline. The absorbance measurements were performed at 510 nm using a UV-Vis spectrophotometer.

**Text S16.** The detection parameters of FT-ICR MS.

The samples were analyzed using a Fourier Transform Ion Cyclotron Resonance Mass Spectrometer (FT-ICR MS, Bruker SolariX, 15T) equipped with an electrospray ionization (ESI) source operating in negative ion mode. Key parameters included continuous sample infusion at 120 μL/h, a capillary inlet voltage of -4.0 kV, an ion accumulation time of 0.06 s, and a mass detection range of 100 - 1600 Da. For each spectrum, 4 M of 32-bit data were acquired, and 300 time-domain transients were co-added to enhance the signal-to-noise ratio 4. Prior to analysis, the instrument was externally calibrated using 10 mmol/L sodium formate. Following acquisition, internal calibration was performed using known molecular formulas of DOM. This calibration process resulted in a mass measurement error consistently below 1 ppm.

**Text S17.** The solid-phase extraction protocols.

The sample pH was adjusted to 2.0 by the dropwise addition of HCl before solid-phase extraction. DOM was subsequently extracted from the sample using an Agilent Bond Elut PPL SPE cartridge (500 mg, 6 mL). The detailed procedure was as follows: (1) The PPL SPE cartridge was activated by sequentially rinsing with 12 mL of methanol and 12 mL of 0.05% HCl. (2) 10 mL of the acidified sample was loaded onto the PPL cartridge at a flow rate of 5 mL min^-1^ to enrich the target compounds. (3) The cartridge was washed with 18 mL of 0.05% HCl to remove salts after sample loading. (4) The PPL cartridge was dried under a stream of nitrogen gas. (5) The retained analytes were eluted with 12 mL of methanol for collection. (6) The collected eluent was evaporated to dryness under a nitrogen stream to completely remove the methanol. (7) The residue was reconstituted in 1 mL of 50% methanol prior to measurement.

**Text S18.** The molecular formula assignment parameters.

Molecular formulas were assigned according to the method reported by Goranov et al. [66]. FT-ICR-MS data spanning the m/z range 100 - 800 was processed. A minimum signal-to-noise ratio of 4 was required for peak inclusion, and a mass accuracy tolerance of 1 ppm was used for formula assignment. Processing was conducted assuming negative ionization mode. Assigned formulae considered the elements C, H (≤ 100 atoms), O (≤ 50 atoms), N (≤ 3 atoms), S (≤ 1 atom) and P (≤ 1 atom). Formula refinement applied elemental ratio constraints (O/C = 0 - 1.2, H/C = 0.33 - 2.25, N/C = 0 - 0.5, S/C = 0 - 0.2, P/C = 0 - 0.1, O/P = 3 - ~, DBE = 0 - 50) following established principles. Subsequent filtering steps included isotopic pattern validation (requiring ^13^C peak S/N ≥ 25), uniqueness checks, Kendrick Mass Defect analysis, compositional plausibility assessment, and mass error evaluation prior to final formula acceptance.

**Text S19.** Machine learning and SHAP analysis

This study employed the XGBoost, Random Forest and LightGBM algorithms to identify molecular features (“Disappeared”, “Resistant”, and “Product”) determining reactivity classifications. The dataset was randomly partitioned into training and testing sets (8:2), and 5-fold cross-validation was applied to balance model bias and variance. Subsequently, Shapley Additive Explanations (SHAP) analysis was utilized to investigate the contribution and impact of molecular features on the model prediction. Positive SHAP values indicate a feature enhances the prediction, while negative values indicate a reduction; the magnitude of the absolute SHAP value reflects the importance of the feature. The specific implementation of the machine learning and SHAP analysis followed the methodology reported by the group of Liu [13].

**Part S2 Supplementary Figure**

**Fig. S1.** (a) The concentration of accumulated •OH upon oxygen exposure following 20 days of oxic incubation. (b) The concentration of accumulated •OH upon nitrogen exposure following 20 days of anoxic incubation.

**Fig. S2.** The concentration of accumulated •OH at different oxygen exposure times following anaerobic incubation for (a) 5 days, (b) 10 days, (c) 15 days and (d) 20 days.

**Fig. S3.** The concentration of dissolved Fe(II) at different oxygen exposure times following anaerobic incubation for (a) 5 days, (b) 10 days, (c) 15 days and (d) 20 days.

**Fig. S4.** The concentration of 0.5 M HCl-extracted Fe(II) at different oxygen exposure times following anaerobic incubation for (a) 5 days, (b) 10 days, (c) 15 days and (d) 20 days.

**Fig. S5.** (a) The initial ORP value at different anaerobic incubation times. (b) The correlation between the •OH accumulated and the ORP value. (c) The correlation between the initial 0.5 M HCl-extracted Fe(II) and the ORP value.

**Fig. S6.** (a) The effect of scavengers on accumulated •OH after 20 days of anaerobic incubation. The effect of (b) BPY and (c) CAT on the concentration of accumulated •OH at different oxygen exposure times after 20 days of anaerobic incubation.

**Fig. S7.** The rarefaction curves of (a) bacterial and (b) fungal communities.

**Fig. S8.** The Chao index of (a) bacterial and (b) fungal communities.

**Fig. S9.** The PCA of (a) bacterial and (b) fungal communities in D20-0, D20-180, D20-300 and Raw soil. (c) The PCA of fungal communities in D20-0, D20-180 and D20-300.

**Fig. S10.** The βNTI of (a) bacterial and (b) fungal communities in D20-0, D20-180, D20-300 and Raw soil.

**Fig. S11.** The Venn diagram of (a) bacterial and (b) fungal communities and the relative abundance of (c) bacterial and (d) fungal communities at the phylum level.

**Fig. S12.** The Venn diagram of (a) bacterial and (b) fungal communities and the relative abundance of (c) bacterial and (d) fungal communities at the genus level.

**Fig. S13.** The phylum-level differential abundance comparison of bacterial communities among D20-0, D20-180 and D20-300 by Kruskal-Wallis H test.

**Fig. S14.** The genus-level differential abundance comparison of fungal communities among D20-0, D20-180 and D20-300 by Kruskal-Wallis H test.

**Fig. S15.** The LDA score plot of LEfSe analysis of bacterial communities among D20-0, D20-180, D20-300 and Raw soil.

**Fig. S16.** The LDA score plot of LEfSe analysis of fungal communities among D20-0, D20-180, D20-300 and Raw soil.

**Fig. S17.** The LDA score plot of LEfSe analysis of fungal communities among D20-0, D20-180 and D20-300.

**Fig. S18.** The Zi-Pi plot of (a) bacterial and (b) fungal ecological networks in D20-0, D20-180 and D20-300.

**Fig. S19.** The correlation heatmap between environmental factors and (a) bacterial and (b) fungal communities at the genus level.

**Fig. S20.** The PCA plot of metabolic profiles in raw soil and anaerobically incubated samples (D20-0, D20-180 and D20-300) with quality control (QC) samples.

**Fig. S21.** The permutation test of the PLS-DA model for metabolic profiles in D20-0, D20-180 and D20-300.

**Fig. S22.** The volcano plots of differential metabolites between (a) D20-0 and D20-180 and (b) D20-180 and D20-300.

**Fig. S23.** (a) The fold changes of representative differential metabolites between D20-0 and D20-180. (b) The KEGG pathway enrichment analysis of differential metabolites in comparisons of D20-0 vs. D20-180.

**Fig. S24.** (a) The fold changes of representative differential metabolites between D20-180 and D20-300. (b) The KEGG pathway enrichment analysis of differential metabolites in comparisons of D20-180 vs. D20-300.

**Fig. S25.** The VIP scores of differential metabolites identified by PLS-DA across D20-0, D20-180, and D20-300.

**Fig. S26.** The KEGG pathway enrichment analysis of differential metabolites identified across D20-0, D20-180, and D20-300.

**Fig. S27.** (a) The DOC variation before and after oxygen exposure. (b) The correlation between the accumulated of •OH and the variation of DOC.

**Fig. S28.** The concentrations of DOC before and after oxidation under conditions of (a) without A-HA, (b) with 180 mg/L A-HA and (c) with 300 mg/L A-HA. The concentration of SOC before and after oxidation under conditions of (d) without A-HA, (e) with 180 mg/L A-HA and (f) with 300 mg/L A-HA.

**Fig. S29.** The EEM fluorescence spectra of DOM extracted from soil suspension with the addition of 0 ((a) and (d)), 180 ((b) and (e)) and 300 ((c) and (f)) mg/L A-HA before and after oxygen exposure treatment following anaerobic incubation for 5 days.

**Fig. S30.** The EEM fluorescence spectra of DOM extracted from soil suspension with the addition of 0 ((a) and (d)), 180 ((b) and (e)) and 300 ((c) and (f)) mg/L A-HA before and after oxygen exposure treatment following anaerobic incubation for 10 days.

**Fig. S31.** The EEM fluorescence spectra of DOM extracted from soil suspension with the addition of 0 ((a) and (d)), 180 ((b) and (e)) and 300 ((c) and (f)) mg/L A-HA before and after oxygen exposure treatment following anaerobic incubation for 15 days.

**Fig. S32.** The EEM fluorescence spectra of DOM extracted from soil suspension with the addition of 0 ((a) and (d)), 180 ((b) and (e)) and 300 ((c) and (f)) mg/L A-HA before and after oxygen exposure treatment following anaerobic incubation for 20 days.

**Fig. S33.** The Van Krevelen diagrams of (a) D20-0-Before, (b) D20-180-Before and (c) D20-180-After. The relative abundance of different samples according to (d) compound classes and (e) element classes. (f) The NOSC-DBE plots of different samples.

**Fig. S34.** The distribution of formula counts in the value of DBE and NOSC (a, c) for samples D20-0-Before and D20-180-Before. The distribution of formula counts in the value of DBE and NOSC (b, d) for samples D20-180-Before and D20-180-After.

**Fig. S35.** (a) The number and proportion of unique and common molecular formulas in the comparison between the D20-0-Before and D20-180-Before. (b) The number and proportion of disappeared, product and resistant molecular formulas in the comparison between the D20-0-Before and D20-180-Before.

**Fig. S36.** (a) The (DBE-O/C)-NOSC plot of molecular formulas commonly present in D20-0-Before and D20-180-Before with color-coded based on the intensity difference. (b) The (DBE-O/C)-NOSC plot of molecular formulas commonly present in D20-180-Before and D20-180-After with color-coded based on the intensity difference.

**Fig. S37.** The (DBE-O/C)-NOSC plot of molecular formulas uniquely present in (a) D20-0-Before and (b) D20-180-Before. (c) The counts percentage across different categories of molecular formulas uniquely present in D20-0-Before and D20-180-Before.

**Fig. S38.** The (DBE-O/C)-NOSC plot of molecular formulas uniquely present in (a) D20-180-Before and (b) D20-180-After. (c) The counts percentage across different categories of molecular formulas uniquely present in D20-180-Before and D20-180-After.

**Fig. S39.** The SHAP value of “Disappeared”, “Resistant”, and “Product” across various characteristic parameters.

**Fig. S40.** The SHAP values of molecular properties calculated by (a) Random Forest and (b) LightGBM.

**Fig. S41.** The degradation trends of (a) DCPA, (b) THM and (c) ATZ at different oxygen exposure times after 20 days of anaerobic incubation.

**Part S3 Supplementary Tables**

**Table S1.** The elemental composition of A-HA.

| **Element Type** | C | H | O* | N | S |
| --- | --- | --- | --- | --- | --- |
| **Elemental Content (%)** | 67.35 | 6.62 | 23.80 | 1.22 | 1.01 |

* The content of oxygen is determined by subtraction.

**Table S2.** The formula counts of D20-0-Before and D20-180-Before in various element and species category.

| **Element** | **A-HA addition (mg/L)** | **Unsaturated and oxidized** | **Unsaturated and reduced** | **Saturated and reduced** | **Saturated and oxidized** | **Total** |
| --- | --- | --- | --- | --- | --- | --- |
| **CHO** | 0 | 64 | 154 | 68 | 36 | 322 |
|  | 180 | 55 | 62 | 58 | 55 | 230 |
| **CHON** | 0 | 303 | 183 | 84 | 108 | 678 |
|  | 180 | 170 | 80 | 40 | 129 | 419 |
| **CHONS** | 0 | 79 | 94 | 21 | 46 | 240 |
|  | 180 | 50 | 79 | 18 | 40 | 187 |
| **CHOS** | 0 | 38 | 66 | 68 | 37 | 209 |
|  | 180 | 24 | 93 | 34 | 26 | 177 |

**Table S3.** The formula counts of D20-180-Before and D20-180-After in various element and species category.

| **Element** | **Oxidation state** | **Unsaturated and oxidized** | **Unsaturated and reduced** | **Saturated and reduced** | **Saturated and oxidized** | **Total** |
| --- | --- | --- | --- | --- | --- | --- |
| **CHO** | Before | 96 | 65 | 67 | 121 | 349 |
|  | After | 54 | 144 | 69 | 24 | 291 |
| **CHON** | Before | 205 | 87 | 43 | 130 | 465 |
|  | After | 265 | 180 | 79 | 121 | 645 |
| **CHONS** | Before | 55 | 88 | 19 | 32 | 194 |
|  | After | 75 | 117 | 20 | 69 | 281 |
| **CHOS** | Before | 23 | 52 | 22 | 21 | 118 |
|  | After | 45 | 131 | 103 | 49 | 328 |

**Table S4.** The list of paired mass distance.

| **Type** | **Paired mass distance** | **Description** |
| --- | --- | --- |
| +2O | 31.989829 | Oxygen addition: (a) Sequential •OH additions to unsaturated/aromatic sites. (b) Further oxidation of initial adducts (alcohols, activated C-H) via radical pathways to form carbonyl/carboxyl groups (+2O-2H). (c) Extensive oxidation, potentially including ring opening (+3O). |
| +3O | 47.984744 |  |
| +2O-2H | 29.974179 |  |
| -CH_2_ | -14.01565 | Dealkylation: Primarily initiated by •OH attack via H-abstraction from C-H bonds in DOM (alkyl, alicyclic structures), generating carbon-centered radicals. These radicals then trigger C-C bond scission via reactions like β-scission. |
| -C_2_H_2_ | -26.01565 |  |
| -C_2_H_4_ | -28.0313 |  |
| -C_2_H_6_ | -30.04695 |  |
| -C_3_H_4_ | -40.0313 |  |
| -C_3_H_6_ | -42.04695 |  |
| -CO_2_ | -43.989829 | Decarboxylation: (a) -CO_2_: Occurs after initial •OH attack leads to oxidation forming unstable precursors (e.g., α-hydroxy/β-keto acids). (b) -C_2_H_2_O_2_, -C_3_H_2_O_2_: Results from oxidative cleavage (e.g., ring opening post-addition/electron transfer, or chain cleavage post-H-abstraction). |
| -C_2_H_2_O_2_ | -58.005479 |  |
| -C_3_H_2_O_2_ | -70.005479 |  |
| -SH_2_ | -33.987721 | Desulfurization: Initiated by •OH attack via H-abstraction (from C-H near S, or S-H), electron transfer (from sulfides/thiols), or addition (ipso-attack on aromatics for -SO_3_). |
| -SO_3_ | -79.956815 |  |
| -NH | -15.010899 | Deamination: Cleavage of C-N bonds (e.g., in amines, amides) likely induced by •OH via H-abstraction (from N-H or adjacent C-H) or electron transfer (from amines), leading to radical intermediates that fragment, releasing -NH. |

**Table S5.** The list of molecular reaction count.

| **Type** | **CHO-CHO** | **CHON-CHO** | **CHON-CHON** | **CHONS-CHON** | **CHONS-CHONS** | **CHONS-CHOS** | **CHOS-CHO** | **CHOS-CHOS** |
| --- | --- | --- | --- | --- | --- | --- | --- | --- |
| +2O | 42 |  | 108 |  | 18 |  |  | 44 |
| +3O | 42 |  | 76 |  | 15 |  |  | 44 |
| +2O-2H | 42 |  | 99 |  | 20 |  |  | 53 |
| -CH_2_ | 69 |  | 146 |  | 33 |  |  | 77 |
| -C_2_H_2_ | 70 |  | 120 |  | 32 |  |  | 80 |
| -C_2_H_4_ | 50 |  | 103 |  | 21 |  |  | 75 |
| -C_2_H_6_ | 57 |  | 82 |  | 12 |  |  | 64 |
| -C_3_H_4_ | 47 |  | 100 |  | 21 |  |  | 62 |
| -C_3_H_6_ | 38 |  | 81 |  | 22 |  |  | 43 |
| -CO_2_ | 96 |  | 176 |  | 25 |  |  | 87 |
| -C_2_H_2_O_2_ | 80 |  | 147 |  | 29 |  |  | 61 |
| -C_3_H_2_O_2_ | 71 |  | 125 |  | 22 |  |  | 55 |
| -SH_2_ |  |  |  | 3 |  |  | 6 |  |
| -SO_3_ |  |  |  | 9 |  |  | 18 |  |
| -NH |  | 17 | 29 |  | 1 | 16 |  |  |
| Total | 704 | 17 | 1392 | 12 | 271 | 16 | 24 | 745 |

**Table S6.** The detailed analytical parameters of p-HBA, DCPA, THM and ATZ.

| **Compounds** | **Mobile phase** | | **Flow rate** | **Detection wavelength** |
| --- | --- | --- | --- | --- |
|  | **A (1‰ acetic acid)** | **B (Methanol)** |  |  |
| p-HBA | 60% | 40% | 0.9 mL/min | 255 nm |
| DCPA | 30% | 70% | 1 mL/min | 250 nm |
| THM | 60% | 40% | 1 mL/min | 250 nm |
| ATZ | 60% | 40% | 1 mL/min | 225 nm |
